# Mechanistic insights into temperature-dependent regulation of the simple cyanobacterial hsp17 RNA thermometer at base-pair resolution

**DOI:** 10.1093/nar/gkv414

**Published:** 2015-05-04

**Authors:** Dominic Wagner, Jörg Rinnenthal, Franz Narberhaus, Harald Schwalbe

**Affiliations:** 1 Institute for Organic Chemistry and Chemical Biology, Center for Biomolecular Magnetic Resonance, Johann Wolfgang Goethe-University, Max-von-Laue-Strasse 7, D-60438 Frankfurt/Main, Germany; 2 Microbial Biology, Ruhr University, Universitätsstr. 150, D-44780 Bochum, Germany

## Abstract

The cyanobacterial hsp17 ribonucleicacid thermometer (RNAT) is one of the smallest naturally occurring RNAT. It forms a single hairpin with an internal 1×3-bulge separating the start codon in stem I from the ribosome binding site (RBS) in stem II. We investigated the temperature-dependent regulation of hsp17 by mapping individual base-pair stabilities from solvent exchange nuclear magnetic resonance (NMR) spectroscopy. The wild-type RNAT was found to be stabilized by two critical CG base pairs (C14-G27 and C13-G28). Replacing the internal 1×3 bulge by a stable CG base pair in hsp17^rep^ significantly increased the global stability and unfolding cooperativity as evidenced by circular dichroism spectroscopy. From the NMR analysis, remote stabilization and non-nearest neighbour effects exist at the base-pair level, in particular for nucleotide G28 (five nucleotides apart from the side of mutation). Individual base-pair stabilities are coupled to the stability of the entire thermometer within both the natural and the stabilized RNATs by enthalpy–entropy compensation presumably mediated by the hydration shell. At the melting point the Gibbs energies of the individual nucleobases are equalized suggesting a consecutive zipper-type unfolding mechanism of the RBS leading to a dimmer-like function of hsp17 and switch-like regulation behaviour of hsp17^rep^. The data show how minor changes in the nucleotide sequence not only offset the melting temperature but also alter the mode of temperature sensing. The cyanobacterial thermosensor demonstrates the remarkable adjustment of natural RNATs to execute precise temperature control.

## INTRODUCTION

Changes in ambient temperature affect the integrity and performance of many cellular structures and processes. Precise temperature perception is therefore essential in particular for those bacterial organisms that occupy environmental niches with varying temperature. Ribonucleicacid thermometers (RNATs) are examples of thermally regulated RNA elements (1) that are located in the 5′-UTR of bacterial messenger RNAs coding for virulence (2–6), cold- (7) and heat-shock genes (8–10). They operate at the post-transcriptional level and alter the accessibility of the ribosome binding site (RBS) to the ribosomal initiation complex in response to temperature changes: in the off-state, a complimentary sequence located in the 5′-UTR sequesters the RBS in a helix, while in the on-state the RBS locally melts and is released to facilitate translation initiation (11). Deviations from Watson-Crick base pairing, that is wobble base pairing found in the fourU motif (2,9,12–14) or non-canonical patterns found in the ROSE element (8,10,15), modulate the stability of the anti-RBS motif. However, variation of the anti-RBS motif alone is not sufficient to fine-tune the temperature regulation of the RBS release needed for the plethora of environmental niches. Structural diversity in the flanking region of the RBS trapping helix has evolved and topologies of naturally occurring RNATs range from short hairpins to complex multi-helix assemblies with critical tertiary interaction (an overview can be found in (11)).

Depending on the environmental challenges, the mode of action of an RNAT is customized for each microbial organism. Bacteria exposed to variations of the ambient temperature need to titrate the amount of chaperones, whereas pathogenic bacteria perceive a temperature shift as successful invasion of a warm-blooded host requiring maximal expression of virulence factors. RNATs may therefore behave either switch-like with a sharp temperature response or as a molecular dimmer, which gradually increases the amount of accessible RBSs over a broad temperature range.

**Figure 1.**
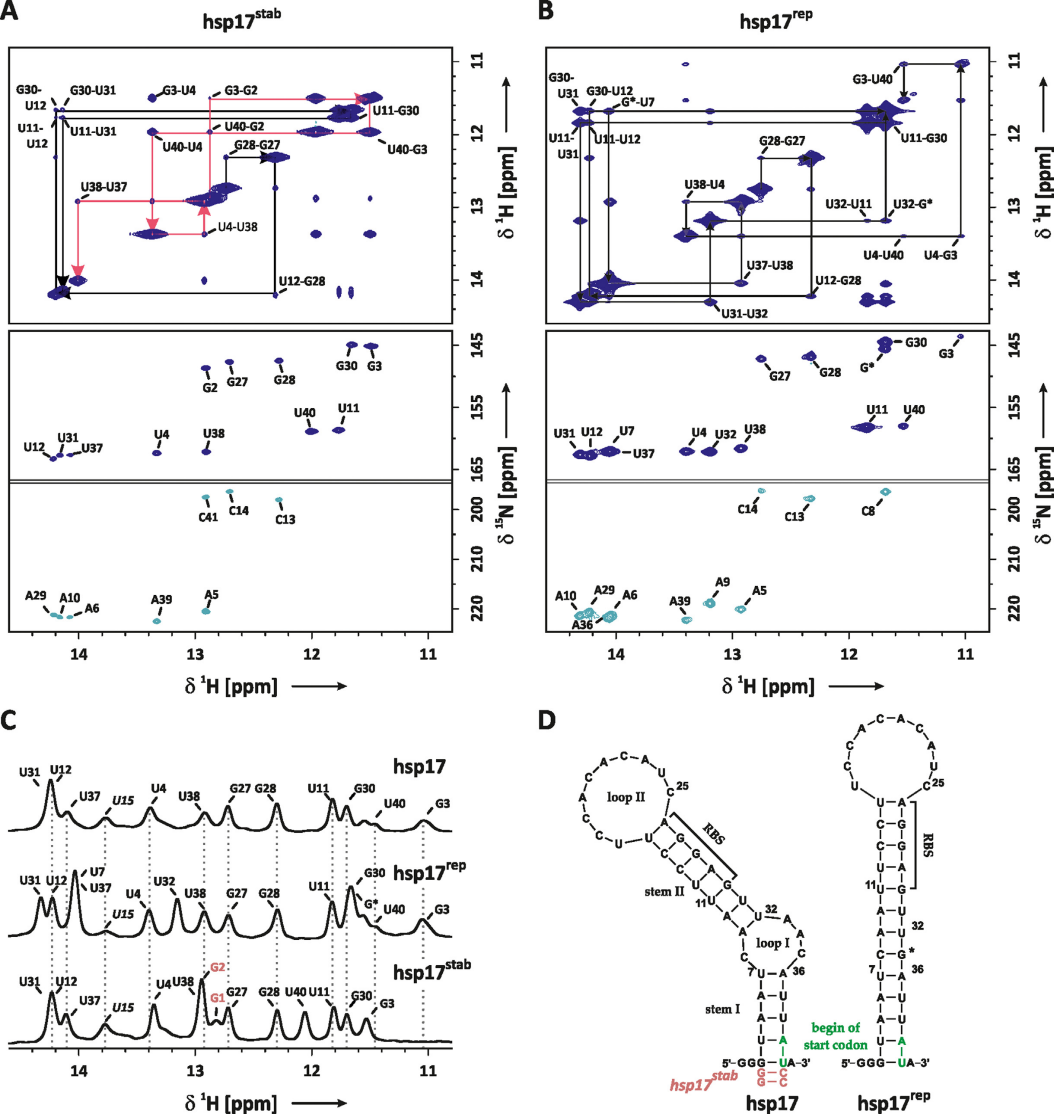
(**A**) Imino proton resonance assignment of the stabilized hsp17^stab^ RNAT: imino region of a ^1^H-^1^H-NOESY spectrum (top) and HNN-COSY spectrum (below). Resonances of G2 and C41 originate from the mutation A40CC, which was introduced to stabilize stem I. Sequential walks for stems I and II are highlighted in red (G2-U38) and black arrows (G27-U31). Both experiments were recorded at *T* = 284 K. (**B**) Imino resonance assignment of the hsp17^rep^ RNAT: imino region of a ^1^H-^1^H-NOESY spectrum (top) and HNN-COSY spectrum (below). Sequential walk is indicated by black arrows (G27-U40). Both experiments were recorded at *T* = 273 K. Base-pair numbering refers to the wild-type sequence shown in (D), G* denotes the mutation AAC(33–35)G. (**C**) Imino proton resonance assignment of hsp17 based on comparison of ^1^H-spectra of hsp17^rep^ and hsp17^stab^ recorded at 274 K. (**D**) Secondary structure of hsp17, hsp17^rep^ and hsp17^stab^ based on NMR resonance assignment. RBS denotes the ribosome binding-site.

Kortmann *et al*. identified the hsp17 RNA thermometer in cyanobacteria (*Synechocystis*) (16). It represents one of the smallest natural RNAT known today. Located in the 5′-UTR of a gene coding for the heat-shock protein 17 (Hsp17), it controls the heat-induced expression of Hsp17 that is vital for the integrity of the photosynthetic apparatus. *Synechocystis* is able to perform photoautotrophic growth over temperatures ranging from 15 to 45°C (17) and functional assays showed that the hsp17 RNAT is able to act as a reversible molecular dimmer (16). Secondary structure determination by enzymatic probing at *T* = 28°C revealed a stable stem-loop sequestering the SD-sequence (Figure 1D) and the first two nucleobases AU of the start codon (16). An asymmetric internal 1×3 bulge further divides the stem into two elements. The RBS is trapped in a helix, but instead of the known tetra-uridine motif found in FourU RNATs, the sequence UCCU sequesters the ribosome-binding site. In other words, the AGGA nucleotides of the RBS are tightly bound. To compensate for this stability and to permit functionality in the physiological temperature range, the immediately adjacent unstructured hairpin consisting of 10 nucleotides is unusually large. The start codon is additionally trapped by stem I, a feature not uncommon for RNA thermometers. A *Synechocystis* hsp17^rep^ mutant, in which the accessibility of the RBS at physiological temperatures is hindered, prevents the expression of the Hsp17 chaperone and elicits a severe phenotype in photosynthetic performance (16).

In this study, we use nuclear magnetic resonance (NMR) spectroscopy to investigate individual base-pair stabilities of the wild type (wt) and the mutant hsp17 RNATs by measuring exchange rates of imino protons with the solvent water at various temperatures. Furthermore, we characterize the temperature dependence of the RNAT global unfolding using circular dichroism (CD) spectroscopy. We derive a detailed molecular mechanism of heat-induced activation of heat-shock proteins required as cellular defence mechanism against heat stress.

## MATERIALS AND METHODS

### RNA preparation

Unlabelled and ^15^N-labelled samples of the hsp17 RNA thermometer constructs with a 3′-fused hammerhead (hsp17 and hsp17^rep^) or 3′-fused hepatitis delta virus ribozyme (hsp17^stab^) were prepared by *in vitro* transcription of linearized plasmid DNA (Supplementary Table S1 for gene sequence) using T7-polymerase as described previously (18). The hsp17^rep^ and hsp17^stab^ constructs carry the mutations AAC(33–35)G and A40CC, respectively. To allow *cis*-cleavage of the 3′-fused hammerhead, we introduced a conservative purine to purine mutation by replacing the single-stranded G nucleotide of the start codon AU(G) located at the 3′ end by AU(A). RNA samples were purified using denaturing polyacrylamide gel electrophoresis (PAGE) (19), subsequently eluted in 0.6 M sodium acetate pH 5.5, desalted using spin concentrators (MWCO 5000 Da) and precipitated in 2% (w/v) LiClO_4_, 80% (v/v) acetone. RNA was dissolved in water at a concentration of 500 μM and folded by denaturation at 95°C for 10 min followed by rapid 1:50 dilution with ice-cold water and subsequent incubation on ice for 30 min. Native 15% PAGE analysis confirmed that NMR samples contained monomeric hairpins (Supplementary Figure S4B).

### CD spectroscopy

Thermal unfolding of the hsp17 RNA thermometer constructs was investigated by CD spectroscopy on a JASCO spectropolarimeter J-810. The melting curves were recorded with a temperature slope of 1°C/min at a wavelength of 260 nm. The samples were measured at a concentration of 20 μM in 5 mM K*_x_*H*_y_*PO_4_, 245 mM KCl pH 6.8 in a cuvette with sample diameter of 1 mm. The melting point *T_m_*, the enthalpy $\Delta H_{\rm unf}$ and entropy $\Delta S_{unf}$ of the melting process and the linear temperature dependence of the CD baseline in the folded and unfolded RNA were determined by fitting the experimental data in *Mathematica* 10 (20) (see Supplementary Figure S3 for details). Using the fitted temperature-dependent baseline, CD data were baseline corrected and normalized to obtain the fraction unfolded (21).

### NMR spectroscopy

NMR experiments were recorded on AV600, AV800, AV900 and AV950 Bruker NMR spectrometers each equipped with a 5 mm cryogenic ^1^H, ^13^C, ^15^N *z*-gradient probe. NMR-spectra were processed using the Bruker TopSpin 2.1/3.1 software and analysed with Sparky 3.1.1.3 (22). All sample contained 100 μM 4,4-dimethyl-4-silapentane-1-sulfonic acid (DSS) as internal NMR standard.

Imino resonances were assigned by standard ^1^H-^1^H-NOESY, ^1^H-^15^N-HSQC and HNN-COSY experiments. See Supplementary Table S2 for details on the experimental parameters. ‘Selective inversion recovery experiments’ were used to measure imino water proton exchange rates at *B*_0_ = 14.1 T (600 MHz). A water selective RE-BURP 180° pulse followed by an incremented delay was placed at the beginning of an ^1^H-NMR or ^1^H,^15^N-HSQC experiment. During this delay a low-power bipolar *z*-gradient was switched on in order to prevent radiation damping (23), which would influence the longitudinal relaxation of the water protons. The inversion recovery profiles of exchangeable imino protons were fitted to the following equation:

(1) \begin{equation*} \frac{{I_{\rm H} \left( {t_{\rm m} } \right)}}{{I_{\rm H} \left( 0 \right)}} - 1 = - 2k_{\rm ex} \frac{{\exp \left( { - R_{1,\rm H} t_{\rm m} } \right) - \exp \left( { - R_{1,\rm W} t_{\rm m} } \right)}}{{R_{1,\rm W} - R_{1,\rm H} }}.\end{equation*}

Here, we assumed that the water magnetization was perfectly inverted by the calibrated RE-BURP 180° pulse. $I_{\rm H} \left( {t_{\rm m} } \right)$ represents the intensities of the imino proton recorded at the inversion recovery mixing time *t*_m_. Note that the imino exchange rate *k*_ex_ obtained from the selective inversion recovery experiment contains a contribution *d* from dipolar cross polarization (24). *k*_ex_ and the longitudinal relaxation rates of the imino proton ($R_{1,{\rm H}}$) and the water proton ($R_{1,\rm W}$) were allowed to adjust freely during the fitting procedure. The temperature within the NMR sample was calculated from the chemical shift of DSS based on external calibration using perdeuterated methanol (details are given in the supplementary information (SI)) (25).

### Thermodynamic analysis of base-pair stability by NMR-detected solvent exchange

The thermodynamic analysis of base-pair stability is based on the measurement of imino exchange rates by selective inversion recovery experiments at varying temperatures and catalyst concentrations and was described in detail previously (12,24). The analysis is briefly recapitulated.

#### Theory

In the open state the labile imino proton of guanine or uracil residues is exposed to solvent water and can exchange with a water proton. The exchange leads to line broadening of the imino proton resonance in ^1^H-NMR spectra and is characterized by the imino proton exchange rate *k*_ex_. If the base pair opens and closes many times before a proton can be exchanged, the base pair reaches an equilibrium (*K*_Diss_) between the hydrogen-bonded conformation and the open state prior to the exchange (EX2 regime). In the EX2 regime, *k*_ex_ only depends on the stability of the base pair and the kinetics (*k*_Tr_) of the proton transfer (26,27):

(2) \begin{equation*} k_{\rm ex} = \frac{{k_{\rm Tr} }}{{1 + 1/K_{\rm Diss} }}.\end{equation*}

The exchange rate is directly related to the temperature dependence of the base-pair opening:

(3) \begin{equation*} K_{\rm Diss} \left( T \right) = {\rm exp}\left( { - \frac{{\Delta H_{\rm Diss} - T\Delta S_{\rm Diss} }}{{RT}}} \right).\end{equation*}

We applied the van't Hoff isotherm and inserted the Gibbs–Helmholtz equation under the assumption that the enthalpy $\Delta H_{\rm Diss}$ and the entropy $\Delta S_{\rm Diss}$ of the base-pair opening is temperature-independent (12). The validity of this assumption is further discussed below. Combining Equations (2) and (3) yields

(4) \begin{equation*} k_{\rm ex} \left( T \right) = \frac{{k_{\rm Tr}}}{{1 + {\rm exp}\left( {\frac{{\Delta H_{\rm Diss} - T\Delta S_{\rm Diss} }}{{RT}}} \right)}}.\end{equation*}

Imino proton exchange is catalysed through external and internal pathways (28): while external catalysis is mediated by small basic molecules (26,27,29) (e.g. OH^−^, HPO_4_^2−^) that are dissolved and have to reside in the local water sphere of the RNA molecule, nucleophilic groups of neighbouring nucleobases (e.g. carbonyl-O and N3 of cytosine, N1 of adenine) adjacent to the observed imino proton are suggested (26,27) to act as internal catalysts. $k_{\rm Tr}$ can therefore be divided into an internal $k_{\rm Tr,int}$ and an external reaction rate $k_{\rm Tr,ext}$:

(5) \begin{equation*} k_{\rm Tr} = k_{\rm Tr,int} + k_{\rm Tr, ext}. \end{equation*}

$k_{\rm Tr,int}$ is related to the enthalpy and entropy of activation $\Delta H^{\rm Tr,int} ,\,\Delta S^{\rm Tr,int}$ through the Eyring equation:

(6) \begin{equation*} k_{\rm Tr,int}\left(T\right) = \frac{{k_B T}}{h}{\rm exp}\left( { - \frac{{\Delta H^{\rm Tr,int} - T\,\Delta S^{\rm Tr,int} }}{{R\,T}}} \right).\end{equation*}

Assuming that access of the catalyst to the nucleobase is not restricted by steric or charged interactions, $k_{\rm Tr,ext}$ can be obtained from the imino proton exchange rate $k_{\rm ex}^{\rm UTP,GTP} \left( T \right)_{c_{\rm HPO_4^{2 - } } }$ of the free mononucleotides UTP/GTP in the presence of the catalyst HPO_4_^2−^:

(7) \begin{equation*} k_{\rm Tr,ext} \left( {c_{\rm cat} ,T} \right) = d_{\rm dif} k_{\rm ex}^{\rm UTP,GTP} \left( T \right)_{c_{\rm HPO_4^{2 - } } }. \end{equation*}

The exchange rates of the free nucleotides were previously determined (24) and the calculation of the diffusion-corrected transition state rate is shown in the SI. Accounting for the dipolar cross polarization *d* (see above) and combining Equations (4–7) yield:

(8) \begin{eqnarray*}&& k_{\rm ex} \left( c_{\rm cat} ,T \right)\\ \nonumber && {=} \frac{{\exp \left( { - \frac{{\Delta H^{\rm Tr,int} - T\,\Delta S^{\rm Tr,int} }}{{R\,T}}} \right) {+} k_{\rm Tr,ext} \left( {c_{\rm cat} ,T} \right)}}{{\frac{h}{{k_{\rm B} T}}\left( {1 {+} \exp \left( {\frac{{\Delta H_{\rm Diss} - T\,\Delta S_{\rm Diss} }}{{R\,T}}} \right)} \right)}} {+} d.\end{eqnarray*}

Equation (8) describes the temperature and concentration dependence of the imino proton exchange rate obtained from selective inversion recovery experiments for a hydrogen-bonded nucleobase in the presence of a catalyst. The stability of individual base pairs can be determined from a temperature series of imino proton exchange rates measured at two concentrations of an external catalyst that meets the EX2 criterion (24).

#### Procedure

Imino exchange rates of the hsp17 constructs were measured at different temperatures at different conditions: (i) in 5 mM K*_x_*H*_y_*PO_4_, pH 6.8, 245 mM KCl and (ii) in 100 mM K*_x_*H*_y_*PO_4_, pH 6.8, 120 mM KCl. The concentration of K^+^ was kept constant at 250 mM in order to prevent modulation of the stability of the RNA through varying cation concentrations (24,30). External catalysis in Equation (8) was calculated (see SI) based on the previously determined transition state enthalpy $\Delta H^{\rm Tr,NTP}$ and entropy $\Delta S^{\rm Tr,NTP}$ of the imino proton exchange for UTP and GTP caused by the catalyst HPO_4_^2−^ (24). The stability of the individual base pairs and their transition states and the respective errors were determined in a Monte Carlo (MC) simulation in which input parameters and datasets at low- and high-catalyst concentration were repeatedly Gaussian noised and globally fitted to Equation (8). Details of the MC simulation and the calculation of the MC errors (31) are given in the SI.

## RESULTS

### Imino proton resonance assignment

To validate previous secondary structure predictions of the hsp17 RNAT (16), imino proton and nitrogen resonances of ^15^N-labeled RNA samples were assigned using a combination of ^1^H-^1^H-NOESY, ^1^H-^15^N-HSQC and HNN-COSY experiments (Figure 1). Here, the HNN-COSY spectrum was used to identify base pairs and their respective connectivities were established by a sequential walk of the imino proton resonances in the ^1^H-^1^H-NOESY (32).

Imino proton–imino proton cross peaks in the ^1^H-^1^H-NOESY spectrum of hsp17^rep^ (Figure 1B, top) could be sequentially connected from G27 to the terminal wobble base pair G3/U40 (base-pair numbering refers to the wild-type sequence). The assignment was aided and confirmed by the base-pair pattern observed in the HNN-COSY spectrum of hsp17^rep^ (Figure 1B, bottom).

The assignment of the natural hsp17 structure was impaired by significant line broadening of several imino resonances that prevented the detection of cross peaks in the ^1^H-^1^H-NOESY and the HNN-COSY spectra (data not shown) even at low temperature (T = 273 K) and low external catalyst concentration (5 mM K*_x_*H*_y_*PO_4_, pH 6.8). Part of the hsp17 assignment could be inferred from hsp17^rep^ based on the frequency and line width of the ^1^H-resonances in the ^1^H spectra (Figure 1C). Differences were found for imino protons adjacent to the mutation AAC(33–35)G preventing the unambiguous assignment of U31, U12 and U37 in hsp17. In order to further assign the missing resonances, a third construct carrying the mutation A40CC (hsp17^stab^) was prepared. The mutation A40CC stabilizes stem I by forming two GC Watson-Crick base pairs at the terminus of the helix leaving the structure of the junction between stem I, loop I and stem II unaltered. Two sequential walks could be identified in the ^1^H-^1^H-NOESY of hsp17^stab^ (Figure 1A, top) even at an increased temperature of *T* = 284 K, suggesting that imino proton exchange was significantly reduced for imino protons in stem I by the mutation A40CC. In conjunction with the base-pair identification by the HNN-COSY experiment (Figure 1A, bottom) the imino proton assignment and the secondary structure of hsp17^stab^ could be established. Comparing the ^1^H-NMR spectra of hsp17^stab^ with the wild-type hsp17 then further allowed the unambiguous assignment of U31, U12, U7 and U37 (Figure 1C). A significantly exchange-broadened resonance at 13.8 ppm was recorded in the ^1^H-NMR spectra of all three constructs. Imino–imino cross peaks originating from this resonance could not be detected in any of the ^1^H-^1^H-NOESY spectra. Judging from its chemical shift (32), the resonance likely belongs to U15, which is located at the base of loop II forming a hydrogen bond in a weak Watson–Crick base pair with A26. The assignment of U15 is confirmed by cross peaks between H2 of A26 and the imino protons of U15 and G27 observed in the imino/aromatic region of the ^1^H-^1^H-NOESY of hsp17 (Supplementary Figure S5). Additional resonances were not detected, indicating that the internal loops are unstructured. The NMR assignments of hsp17 and hsp17^rep^ are in agreement with the secondary structure models that were previously determined by chemical and enzymatic probing at 28°C (16).

Note that the imino resonances of U15, G27, G28, U12 and U11 in the RBS and U4 and U38 are very similar in hsp17 and hsp17^rep^ (Figure 1C). Since imino chemical shifts are very sensitive to changes in the base-pair conformation, we conclude that the conformation of these nucleobases is not altered in hsp17^rep^. However, U31 and U37 exhibit chemical shift perturbations of their ^1^H imino resonances, indicating that the base pairs A10-U31 and A6-U37 experience an alteration of their base-pair geometry and stacking interactions resulting from the AAC(33–35)G mutation.

### Temperature dependence of imino proton water exchanges rates

**Figure 2.**
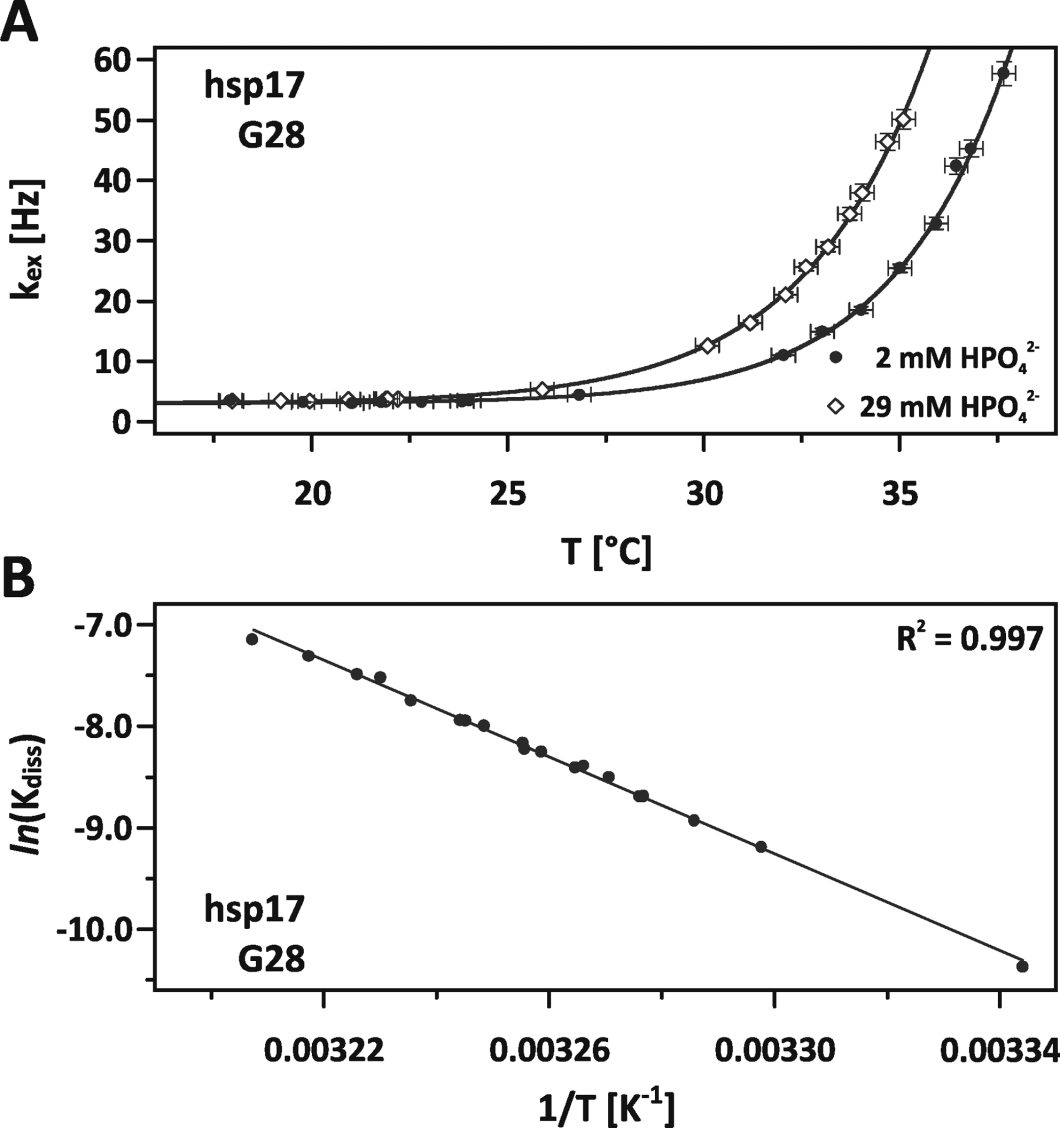
(**A**) Fit of imino proton exchange rates *k*_ex_ of G28 (hsp17) against varying temperatures and catalyst concentrations of 2 mM (circles) and 29 mM (diamonds) HPO_4_^2−^. Error bars represent standard errors. (**B**) Van't Hoff plot of the base-pair opening of C13-G28 calculated from imino exchange rates shown in (A) and linear interpolation.

Imino exchange rates *k*_ex_ measured for hsp17 and hsp17^rep^ obtained at 2 and 29 mM HPO_4_^2−^ and varying temperatures were fitted against the catalyst concentration and the temperature. Figure 2A shows the exchange rates of the G28 imino resonance in hsp17.

At low temperature, exchange rates are dominated by a constant contribution from dipolar cross relaxation (24). With raising temperature, the base-pair stability is reduced and the population of the open state is increased leading to a significantly higher exposure of the labile imino proton to internal catalytic groups and the external catalyst HPO_4_^2−^. As a consequence the exchange rate increases. At higher catalyst concentration, the chance of a successful proton transfer is increased and the line broadening at 29 mM HPO_4_^2−^ is shifted to lower temperatures. The interpolations obtained from the MC-simulation fit remarkably well, in particular a systematic deviation from the fit indicating a change of heat capacity is not observed. The van't Hoff plot in Figure 2B shows the temperature dependence of the dissociation constant *K*_diss_. The data exhibit a high linear correlation (*R*^2^ = 0.997) with no apparent curvature justifying our assumption to treat the enthalpy and entropy as temperature independent (33).

**Figure 3.**
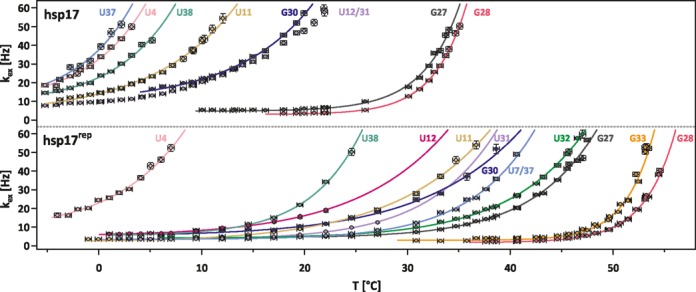
Imino proton exchange rates *k*_ex_ of indicated nucleobases in hsp17 and hsp17 ^rep^ at varying temperatures and a catalyst concentration of 29 mM HPO_4_^2−^. Error bars represent standard errors. In hsp17, the temperature profile of U12/U31 was not fitted.

Imino resonances of hsp17 and hsp17^rep^ exhibited differential line broadening in the course of the temperature series and the temperature dependence of the *k*_ex_-rates varied significantly among individual nucleobases. This variation necessitated the measurement of the exchange rates over a broad range of temperatures from −5 to 55°C. A total of 191 selective inversion experiments were conducted and 783 exchange rates were included in the analysis. The exchange rates of hsp17 and hsp17^rep^ at 29 and 2 mM HPO_4_^2−^ are shown in Figure 3 and Supplementary Figure S2, respectively. Imino resonances in stem I (hsp17) experienced severe exchange at the freezing point of water, while resonances of nucleobases in stem II only began to broaden at significantly elevated temperatures.

On the contrary in hsp17^rep^, the line broadening profiles of all imino resonances were shifted to higher temperatures compared to hsp17, indicating a stabilization of all base pairs in the helix introduced by the AAC(33–35)G mutation. It is remarkable that in hsp17^rep^ the temperature characterizing the onset of base-pair opening for different base pairs varied between −2 and 48°C.

Terminating base pairs are usually weak, as they have only one stacking interaction, and can be detected in ^1^H-NMR spectroscopy only at low temperatures because of severe exchange broadening. In line with this notion, imino exchange of the terminal wobble base pair G3-U40 and the loop flanking nucleobases U15 (hsp17 and hsp17^rep^) and U7, U32 (hsp17) was already severe in the high-catalyst buffer at temperatures below 0°C. Freezing of the sample prevented the detection of *k*_ex_*-*rates at even lower temperatures and *k*_ex_*-*rates could not be measured over a sufficiently broad temperature range that would allow an accurate analysis of the base-pair stability. Spectral overlap was observed for U12, U31 (hsp17) and U7, U37 (hsp17^rep^) in both the ^1^H and the ^15^N dimensions over the course of the temperature series. Fitting the inversion recovery profiles of the overlapping resonances to individual imino protons failed due to non-convergence. We fitted average *k*_ex_-rates from the inversion recovery profile of the U7/U37 and the U12/U31 peaks. The respective fits did not exhibit significant deviations from the inversion recovery data supporting the assumption of comparable exchange behaviour. The exchange rates of U12/U31 show a similar temperature dependence as G30, revealing that at least one of the nucleobases is of comparable stability as G30 in hsp17. Since U7, U37 (hsp17^rep^) are base-pair neighbours, we fitted the imino exchange rates under the assumption that both base pairs have a comparable stability and determined an average base-pair stability.

### Thermodynamic stability of the base-pair opening

Enthalpy ($\Delta H_{\rm diss}$), entropy ($\Delta S_{\rm diss}$) and Gibbs energy ($\Delta G_{\rm diss} \left( {T = 20^ \circ {\rm C}} \right)$) as well as the internal transition state parameters of the individual base-pair opening were determined for the RNAT hsp17 and hsp17^rep^ from the temperature dependence of the imino exchange rates obtained at varying temperatures and catalyst concentrations. The results are summarized in Tables 1 and 2 and Supplementary Tables S4 and S5. The magnitude of observed enthalpies and entropies spread from 38.8 to 277.8 kJ/mol and 56.7 to 781.2 J/(K mol), respectively. These values are within the range of published results on the individual base-pair opening of RNA duplexes and hairpins (12,24,34). On average, GC base pairs exhibited larger values than AU base pairs in both RNATs. The enthalpy and entropy of the GU wobble base pair in hsp17 and hsp17^rep^ were found to be comparable or even lower than for AU base pairs. Gibbs energies $\Delta G_{\rm diss} \left( {T = 20^\circ {\rm C}} \right)$ of AU and wobble GU base pairs ranged from 10.1 to 26.5 kJ/mol. GC base pairs were on average more stable at 20°C with $\Delta G_{\rm diss} \left( {T = 20^\circ C} \right)$-values spreading from 26.2 to 48.8 kJ/mol.

**Table 1.** Fit results of the individual base-pair stabilities in hsp17

hsp17	Δ*H*_diss_	ΔΔ*H*_diss_	Δ*S*_diss_	ΔΔ*S*_diss_	Δ*G*_diss_ (*T* = 20°C)	ΔΔ*G*_diss_ (*T* = 20°C)
	[kJ/mol]	[kJ/mol]	[J/(mol K)]	[J/(mol K)]	[kJ/mol]	[kJ/mol]
G3/U40	n.d.	n.d.	n.d.	n.d.	n.d.	n.d.
U4	64.3	8.5	182.6	30.9	10.8	0.6
U38	71.2	10.8	200.9	39.3	12.3	0.7
U37	65.9	12.8	190.2	46.9	10.1	1.0
U7	n.d.	n.d.	n.d.	n.d.	n.d.	n.d.
U32	n.d.	n.d.	n.d.	n.d.	n.d.	n.d.
U31	/	/	/	/	/	/
U11	38.8	3.7	82.9	13.1	14.5	0.2
G30	46.1	3.7	100.7	12.9	16.6	0.2
U12	/	/	/	/	/	/
G28	193.5	23.7	561.9	77.5	28.8	1.0
G27	148.4	20.4	416.1	67.1	26.2	0.8
U15	n.d.	n.d.	n.d.	n.d.	n.d.	n.d.

n.d., not determinable due to low base-pair stability and resultant exchange broadening of the imino resonance. U31 and U12 could not be analysed due to spectral overlap. ΔΔ*H*_diss_, ΔΔ*S*_diss_ and ΔΔ*G*_diss_ (*T* = 20°C) represent the Monte Carlo error.

**Table 2.** Fit results of the individual base-pair stabilities in hsp17^rep^

hsp17^rep^	Δ*H*_diss_	ΔΔ*H*_diss_	Δ*S*_diss_	ΔΔ*S*_diss_	Δ*G*_diss_ (*T* = 20°C)	ΔΔ*G*_diss_ (*T* = 20°C)
	[kJ/mol]	[kJ/mol]	[J/(mol K)]	[J/(mol K)]	[kJ/mol]	[kJ/mol]
G3/U40	n.d.	n.d.	n.d.	n.d.	n.d.	n.d.
U4	53.0	6.0	137.8	21.6	12.6	0.4
U38	102.1	8.1	283.0	27.5	19.2	0.2
U7/U37	86.9	8.0	206.5	26.2	26.4	0.4
G*	277.8	19.2	781.2	59.3	48.8	1.9
U32	70.0	4.3	148.5	14.1	26.5	0.3
U31	79.7	12.6	185.7	42.7	25.3	0.3
U11	43.0	3.6	73.8	12.2	21.3	0.2
G30	45.8	4.6	82.9	15.3	21.5	0.2
U12	38.8	7.5	56.7	25.7	22.4	0.2
G28	232.5	14.4	637.5	44.4	45.6	1.4
G27	95.9	4.2	232.4	13.7	27.8	0.3
U15	n.d.	n.d.	n.d.	n.d.	n.d.	n.d.

n.d., not determinable due to low base-pair stability and resultant exchange broadening of the imino resonance. ΔΔ*H*_diss_, ΔΔ*S*_diss_ and ΔΔ*G*_diss_ (*T* = 20°C) represent the Monte Carlo error. Nucleobase numbering of hsp17^rep^ is based on hsp17, G* denotes the mutation AAC(33–35)G.

#### Stability of terminating base pairs

^1^H-NMR spectra revealed that the terminating base pairs form hydrogen bonds at low temperatures. As discussed above, exchange broadening of their respective imino proton resonances was severe and *k*_ex_-rates could not be obtained, indicating that the base pairs are already significantly weakened. We conclude that values obtained for U37-A6, which is the weakest base pair according to Tables 1 and 2, serve as an upper limit for the stability values of the terminating base pairs.

#### Impact of AAC(33–35)G mutation

**Figure 4.**
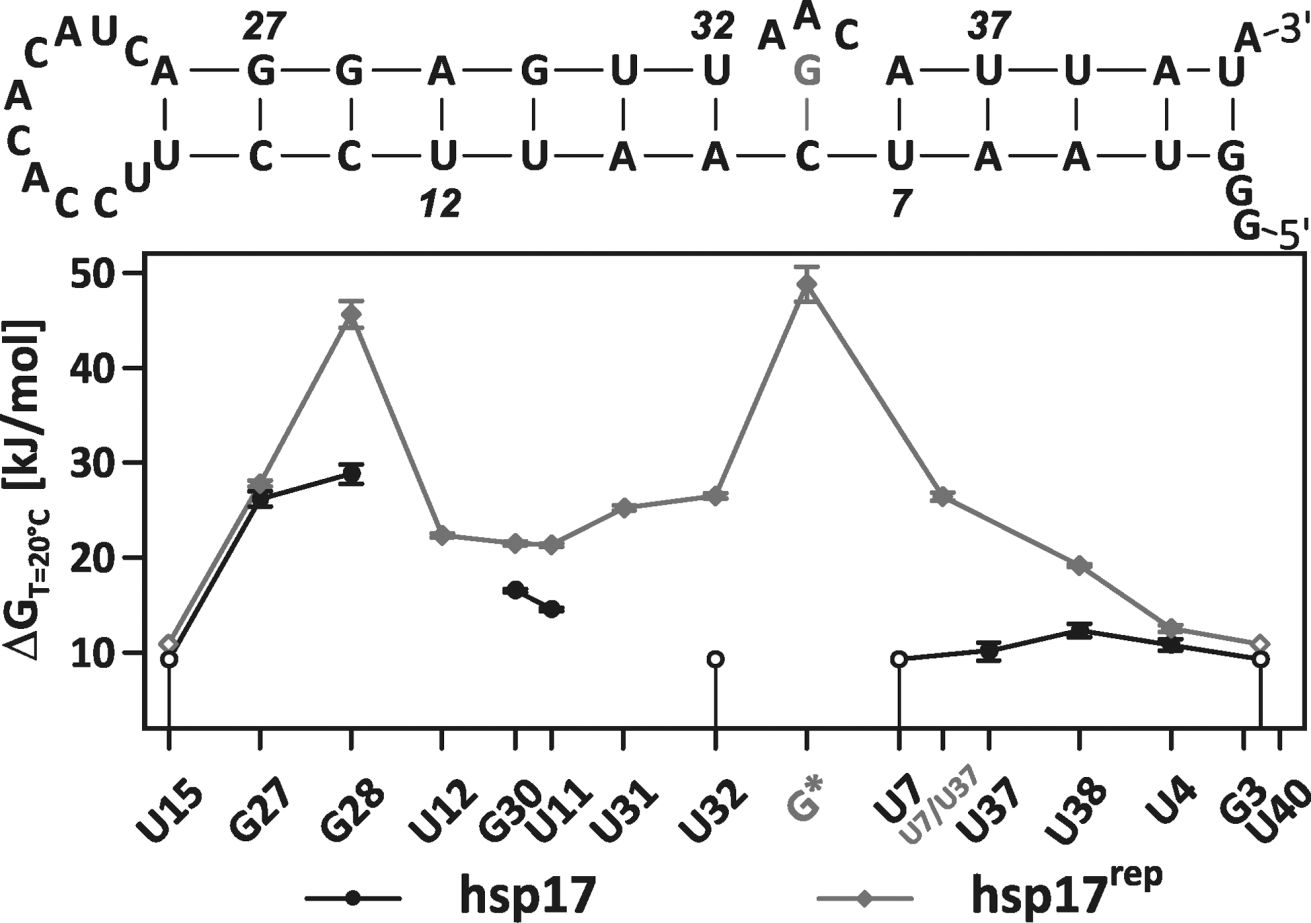
Gibbs energies at 20°C of the uncorrelated base-pair opening in hsp17 (circles) and hsp17^rep^ (diamonds). Gibbs energies of U12 and U31 in hsp17 could not be determined due to spectral overlap. An averaged Gibbs energy was determined for U7 and U37 in hsp17^rep^. Imino resonances of terminally located nucleobases experienced significant line broadening and the respective base-pair stabilities are given as upper limits in open circles/diamonds (see the main text).

The AAC(33–35)G mutation replaces the internal 1×3 bulge forming the extraordinarily stable G*-C8 base pair with $\Delta \Delta G_{\rm diss} \left( {T = 20^\circ C} \right) =$ 48.8 kJ/mol. Its incorporation has a profound impact on the stability of the RNAT: all base pairs in hsp17^rep^ experience an increase in Gibbs energies of the adjacent bases (Figure 4). The stability of U32 and U7 in hsp17 is estimated to be below 10.1 kJ/mol and their counterparts in hsp17^rep^ exhibit $\Delta G_{\rm diss}$-values of 26.5 kJ/mol and 26.4 kJ/mol. The stabilization is not restricted to the nearest neighbours U32 and U7, but is transmitted to more remotely located base pairs. By comparison of the temperature dependence of the imino exchange rates of the double peak U31/U12 (see above), we argue that their Gibbs energies at 20°C are comparable to G30. While the stabilization levels off at the terminus of the hairpin, the centre of the RBS (G28-C14) experiences an unexpected increase in Gibbs energy of $\Delta \Delta G_{\rm diss} \left( {T = 20^\circ \rm C} \right) =$ 16.8 kJ/mol rendering it similarly stable as G*-C8.

**Figure 5.**
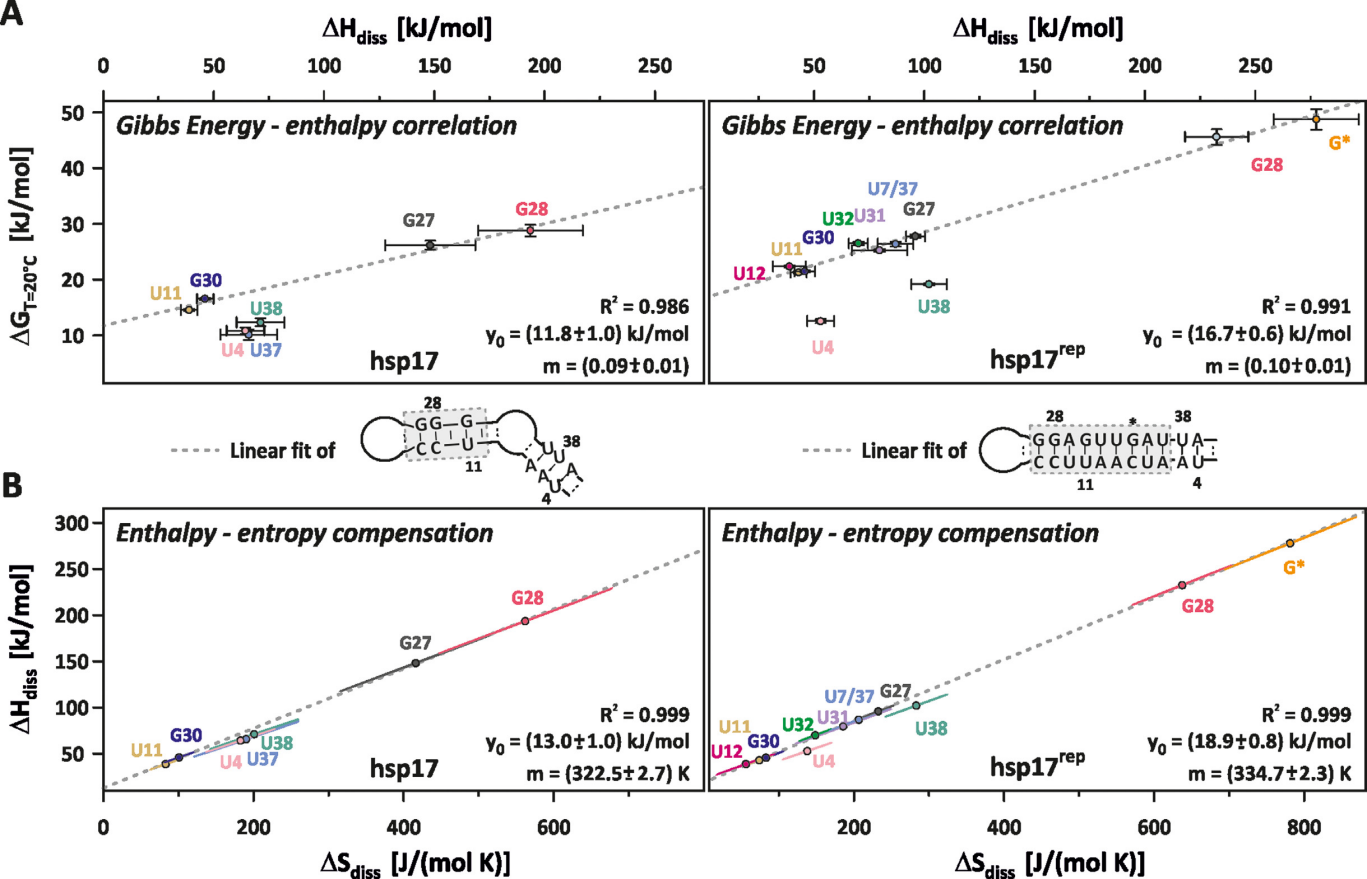
(**A**) Gibbs energy–enthalpy correlation and (**B**) enthalpy–entropy compensation in hsp17 (left) and hsp17^rep^ (right). Correlations of highlighted nucleobases (dashed box) were fitted according to the linear relationship $y\left( x \right) = m*x + y_0$ (dashed line). Error bars in (A) represent the Monte Carlo errors, ellipses in (B) are the confidence ellipses at the one σ-level, which describe the distribution of the Δ*H* and Δ*S* pairs obtained from the Monte Carlo simulation. Errors for the linear fit represent standard deviations obtained from linear regression.

No clear trend emerges for the enthalpy and entropy values in hsp17 and hsp17^rep^ (Tables 1 and 2, Figure 5). For G28, entropy and enthalpy of the base-pair dissociation increase concomitantly in hsp17^rep^, whereas the respective values decrease for G27. For G30 and U11, enthalpy remains nearly constant and entropy decreases.

### Enthalpy–entropy compensation

The enthalpy and entropy for the individual nucleobases are shown in Figure 5. With exception of the terminally located U4 and U38, enthalpy values are linearly correlated to the Gibbs energy values for all nucleobases in hsp17^rep^ (Figure 5A). Albeit a marginally smaller correlation coefficient, nucleobases in stem II of hsp17 also exhibit an unambiguous linear correlation. By virtue of the Gibbs–Helmholtz equation, Gibbs energy–enthalpy correlation implies the enthalpy–entropy compensation (EEC) shown in Figure 5B for the same nucleobases. U4, U38 and U37, which are located in stem I of hsp17, appear to exhibit a second EEC. However, the spread of $\Delta H_{\rm diss}$, $\Delta S_{\rm diss}$-values is too small compared to the respective errors to unambiguously infer their correlation and a linear fit of the respective values was omitted. The slope *m*_EEC_ and the offset *y*_0_ of the observed EEC hold the units of temperature and energy, respectively. EEC is regarded as a common property of water-solute interactions (35) and found in a variety of chemical systems (12,24,34,36–39). The slope *m*_EEC_ is called the compensation temperature *T*_c_ and its value is commonly assumed to depend on solute–solvent interactions. On the contrary, the offset is determined by interactions within the solute. *T*_c_ and the offset *y*_0_ amount to 49°C and 13.0 kJ/mol for hsp17 and to 61.6°C and 18.9 kJ/mol for hsp17^rep^.

Interestingly, not all base pairs within hsp17^rep^ obey the EEC: U4 and U38 (2 and 3 nt away from the terminus) are not involved in the EEC, albeit they are within the same helix. Such deviations of the EEC were previously observed for base pairs near the terminus of an RNA helix and interpreted as a specific fraying effect due to increased motional freedom (34).

The existence of an observed EEC and the physical meaning of the compensation temperature have been a controversial topic for a long time (36,40,41). Much of the debate originates from the statistical compensation effect (42) and its misinterpretation as a hidden physical process (see SI for a more detailed description). The statistical compensation effect can be seen in Figure 5B: each error ellipse is very narrow and indicates a high correlation of $\Delta H_{\rm diss}$ and $\Delta S_{\rm diss}$-values obtained from the MC-simulation. However, the error ellipses of different $\Delta H_{\rm diss}$, $\Delta S_{\rm diss}$-pairs are well separated from each other, showing that the fitted EEC does not arise from statistical error compensation. Statistical tests were proposed (36,43) and their applications to the systems studied here confirm the observed EEC for hsp17 and hsp17^rep^ (Figure 5) and the validity of the derived compensation temperatures (details of the statistical test are given in the SI).

### Global unfolding of the RNAT

**Figure 6.**
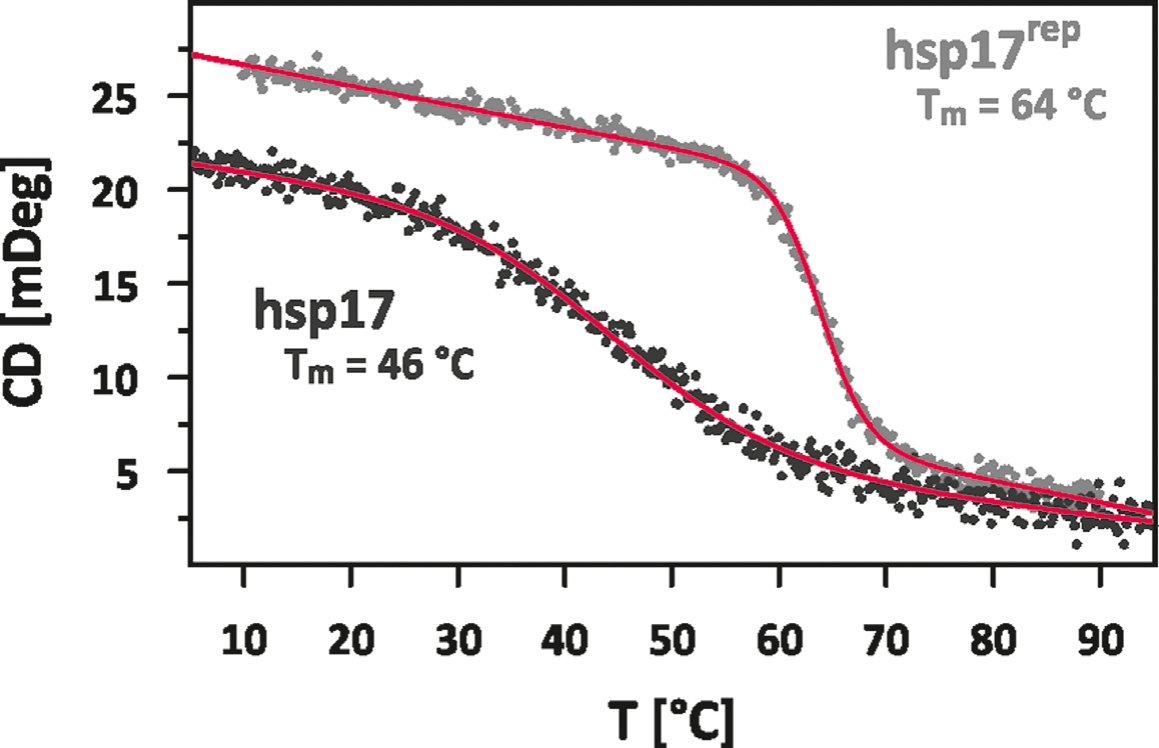
Temperature-induced unfolding curves monitored by CD-spectroscopy of hsp17 (black) and hsp17^rep^ (grey). Parameters of the baseline fit are given in Supplementary Figure S3.

Figure 6 shows the melting curves of hsp17 and hsp17^rep^ obtained from the temperature dependence of the CD-spectrum. hsp17^rep^ unfolded at a melting point of *T*_m_ = 64°C in a highly cooperative manner ($\Delta H_{\rm unf} =$431 kJ/mol, $\Delta S_{\rm unf} =$1278 J/(K mol)). On the contrary, hsp17 exhibited a broad unfolding transition. Extensive line broadening at low temperatures and differences in stability and in the EEC suggest that melting of stem I precedes unfolding of stem II. Short helices composed of only few weak AU base pairs are predicted to undergo a broad unfolding transition (44) indicating that melting of stem I can in principle be uncorrelated to and overlap with the melting of stem II. Yet, the melting curve did not reveal two distinct transitions and the data could be fitted to a two-state model with an apparent melting temperature of *T*_m_ = 46°C ($\Delta H_{\rm unf} =$104 kJ/mol, $\Delta S_{\rm unf} =$326 J/(K mol)) (see Supplementary Figure S3 for details of the fit). Stabilizing the terminus of hsp17 by two GC base pairs (hsp17^stab^) raises the apparent melting temperature by 5°C and increases the unfolding cooperativity significantly ($\Delta H_{\rm unf} =$282 kJ/mol, $\Delta S_{\rm unf} =$870 J/(K mol), Supplementary Figure S3E). The fractions folded of hsp17 and hsp17^stab^ are overlaid in Supplementary Figure S3F: the unfolding of hsp17 is broader both at lower and higher temperatures than the melting point, and stems I and II in hsp17 must exhibit a significantly reduced cooperativity compared to both mutants. We can therefore conclude that the internal loop and the weak stem I broaden the temperature response of the RBS release of the wild-type RNAT.

## DISCUSSION

The hsp17 RNAT is a thermally regulated RNA element that controls the expression of the Hsp17 chaperone in the cyanobacterium *Synechocystis*. Expression studies *in vivo* showed (16) that hsp17 functions as molecular dimmer, which enables cyanobacteria to titrate the appropriate amount of chaperones required to maintain protein and membrane homeostasis at an elevated temperature. Here, we investigated how such cellular function can be explained on the molecular level by investigating the stability of the hsp17 RNAT and a stabilized mutant hsp17^rep^ at a base-pair resolution and by determining the enthalpy, entropy and Gibbs energy at 20°C of the uncorrelated base-pair opening.

We report that the base-pair stabilities vary significantly within hsp17. In stark contrast to many other RNATs, two CG base pairs within the RBS formed the most stable base pairs in the entire RNAT. Base pairs in stem I, which trap the first two nucleotides of the start codon, exhibited significantly lower stabilities. Upon substitution of the internal bulge separating stems I and II with a CG base pair, stabilization was observed for all base pairs with an extraordinary increase of $\Delta \Delta G_{\rm diss} \left( {T = 20^\circ \rm C} \right) =$ 16.8 kJ/mol for G28 forming the central part of the RBS. Enthalpy and entropy of the individual base pairs were found to vary significantly for both hsp17 and hsp17^rep^.

### Nearest and non-nearest neighbour effects stabilize hsp17^rep^

Based on the approach of Searle and Williams (45) to partition the free energy contributions to the formation of single-strand RNA helices, energetic contributions to base-pair stability can be disentangled into the hydrophobic effect, internal rotor motions of the nucleotide, hydrogen bonds and stacking interactions (ignoring tertiary interactions unlikely to be prominent in the short hsp17 thermosensor).

In the closed conformation, the nucleobase is stabilized by hydrogen bonds with its base-pair partner. Based on a study investigating free energy increments upon replacement of guanine with inosine in duplex RNA molecules (46) it is estimated that on average each hydrogen bond contributes about 1 kcal/mol (4.2 kJ/mol) to the base-pair thermal stability (47) making GC base pairs (three hydrogen bonds) more stable than AU and GU base pairs (two hydrogen bonds). However, counting the mere number of hydrogen bonds is not sufficient to explain the observed differences in stability, e.g. U32 (hsp17^rep^) is with $\Delta G_{\rm diss} =$(26.5 ± 0.3) kJ/mol at *T* = 20°C equally stable as G27 (hsp17) with $\Delta G_{\rm diss} =$(26.2 ± 1.0) kJ/mol. As can be seen from the variations of the enthalpy and entropy values, stacking among base pairs and interactions of the nucleobases with the hydration shell (solute-solvent interaction) greatly modulate the stability.

Stacking interaction summarizes the balance of electrostatic interaction, short-range repulsion and London dispersion attraction of the coaxially stacked nucleobases (48). The magnitude of stacking energy results from the specific geometry of the intra- and intercatenar base-pair overlap and is influenced by solvent screening effects (48). In fact, this observation forms the basis of the nearest-neighbour models (NNM, 32,33), which assume that the stability of RNA helices is determined locally: the stacking energy of a given base pair depends on its adjacent base pairs and the resulting stability of the RNA helix is determined on the sequence of base-pair steps.

In hsp17^rep^, the internal 1×3-bulge is replaced by a remarkably stable CG base pair. As one might expect, the adjacent base pairs experience an increase in stability, which can be explained by such neighbour effects: the newly introduced base pair C8-G* induces a stacking interaction with U7-A36 and A8-U32 stabilizing both base pairs. The stabilization is not restricted to the nearest neighbours in hsp17^rep^, but extends to the base pairs on either side of C8-G*. Judging from their chemical shift perturbations (Figure 1C), U31 and U37, which are next in the helix of hsp17^rep^, experience a conformational change of their base-pair geometries as a result of the AAC(33–35)G mutation. A restriction of base-pair mobility and an altered stacking interaction are then likely to concomitantly confer stabilization to U31 and U37 in hsp17^rep^. Somewhat unexpectedly, G28 and G27, which are located 5 and 6 nt away from the AAC(33–35)G mutation and form the centre of the RBS, are also stabilized. Interestingly, the amount of stabilization is considerably different for the two base pairs: while the decrease in enthalpy for C14-G27 is nearly fully compensated by a loss of entropy resulting in a marginal stabilization of $\Delta \Delta G_{\rm diss} \left( {T = 20^\circ \rm C} \right) =$ 1.6 kJ/mol, C13-G28 experiences an extraordinary stabilization of $\Delta \Delta G_{\rm diss} \left( {T = 20^\circ \rm C} \right) =$ 16.8 kJ/mol that arises from a gain in favourable binding enthalpy of $\Delta \Delta H_{\rm diss} =$39.0 kJ/mol outweighing the entropic cost $T_{20^\circ \rm C} *\Delta \Delta S_{\rm diss} =$ 22.2 kJ/mol of the more stable base pair. The conservation of ^1^H imino resonances reveals that the base pairing interface remains unchanged for G27 and G28 in hsp17^rep^ compared to hsp17. NNM predicts (49,50) that the G28 step confers a slightly larger stabilization of −1.1 kJ/mol to the global helix stability than the G27 step. Whereas the base-pair stability differences of G27 and G28 observed in hsp17 (Table 1) are consistent with the NNM prediction, the results in hsp17^rep^ underline the deviation from nearest-neighbour models. As marked differences in the melting temperature and the global unfolding enthalpy/entropy indicate, such deviations from NNM predictions become also manifest in the global unfolding process (we discuss the relationship of the individual base-pair stabilities and the global stability of the RNA in the SI).

Non-nearest neighbour interactions leading to an unexpected global stabilization were observed in NNM studies of RNA helices containing bulge loops (51,52), internal 1×2 bulges (53) and hairpin tetraloops (54). Additionally, we previously identified a long-range stabilization for a stabilized mutant of the *Salmonella* fourU RNAT (12): here, a AG mismatch was replaced by a CG base pair. As a consequence, another GC pair located 6 nt away from the mutation was stabilized by $\Delta \Delta G_{\rm diss} \left( {T = 20^\circ \rm C} \right) =$ 9.8 kJ/mol (55). None of the aforementioned studies could reveal the physical/chemical origin for the non-nearest neighbour interactions. However, we implicated the hydration network around the RNA as a possible mediator in the long-range effect (12).

In an RNA A-helix each base pair is hydrated by up to 21–22 water molecules (56–58) and the water molecules were found to form a regular network in the major and minor groove (59) with a significantly reduced mobility compared to bulk water. Recent studies (60–64) suggest that water dynamics in the hydration shell of nucleic acids is highly sequence- and structure-dependent, indicating a heterogeneous entropy distribution within the hydration shell of RNA helices. Since the hydration shell forms a network, the dynamics and entropy of the water molecules surrounding neighbouring base pairs must be coupled as well. Imino exchange spectroscopy is sensitive to the hydration shell: once a base pair swings out through the minor or major groove (65), the nucleobase intrudes into the hydration shell, disrupts the water network and forms new hydrogen bonds with water molecules. The spread of observed entropies in hsp17 and hsp17^rep^ indicates that each base pair adopts a specific balance of motional freedom and entropy of water. Interestingly, this balance is linked among the base pairs highlighted in Figure 5 through the EEC. Here, increased entropy of the base-pair opening comes along with a larger enthalpy and vice versa (Figure 5B). Both effects act in a compensatory manner and attenuate the span of observable Gibbs energies.

In light of the observed EEC, it is reasonable to argue that the non-nearest neighbour effects result from the same mechanism that contributes to the EEC: the closure of the 1×3 bulge by a stable CG base pair suppresses fraying near the internal ends of the helix (increased conformational entropy) and induces more favourable stacking interactions at the neighbouring base pairs. This stiffening leads to more favourable dynamics throughout the hydration shell allowing greater ordering of water molecules and more favourable hydrogen bonding geometries. It must be noted that the precise mechanism resulting in a linear EEC remains unknown. Yet, the here presented findings and previous studies from us (12,24) and Chen and Russu (34) suggest that linear EEC of the uncorrelated base-pair opening is a common feature observed for nucleobases joined in a helical RNA and reflects the thermodynamic coupling of the base pairs and the hydration shell.

### Global unfolding

**Figure 7.**
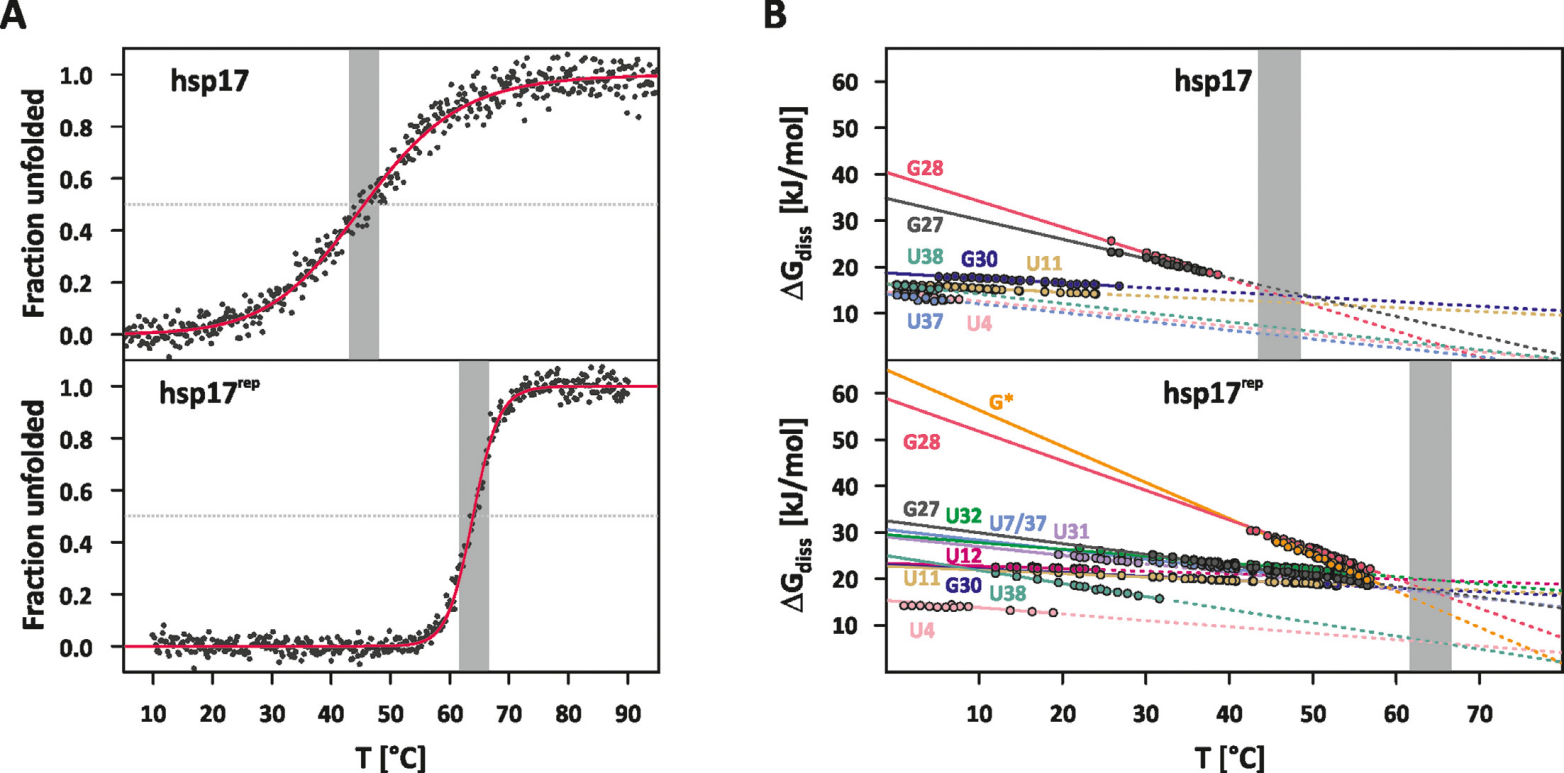
(**A**) Temperature dependence of the fraction unfolded obtained from CD-melting curves of hsp17 (top) and hsp17^rep^ (bottom). (**B**) Temperature dependence of Gibbs energies of the base-pair stability. Δ*G*_diss_ values (circles) were calculated from the imino exchange rates obtained at catalyst concentrations of 2 and 29 mM HPO_4_^2−^ using internal and external transition state parameters. Dashed lines are extrapolations of the Gibbs energy assuming the nucleobase pair is still formed. Grey-shaded area in (A) and (B) highlights the temperature range *T*_m_ ± 2.5°C.

EEC implies that all participating base pairs are equally stable at the compensation temperature. This can be verified from the extrapolated temperature dependence of the Gibbs energies (Figure 7B). Within the margin of error, the Gibbs energies of the base pairs involved in the EEC coincide at *T* = *T*_c_. Interestingly, the melting points of wt (*T*_m_ = 46°C) and mutant RNAT (*T*_m_ = 64°C) (Figure 7A) are very close to the compensation temperatures *T*_c_ = (49.4 ± 2.7)°C and *T*_c_ = (61.6 ± 2.3)°C, respectively.

The correspondence *T*_c_ ≈ *T*_m_ was observed before in helical RNA and DNA exhibiting an EEC and was attributed to a cancellation of stabilizing effects induced by the hydration shell network on uncorrelated base-pair opening at the melting point (12,24). Here, the helix is equally stable as the unfolded conformation lacking most of the ordered intrinsic interactions (solute–solute). According to Searle and Williams (45), stacking interactions are the dominating intrinsic interactions in the helical conformation, which are compensated by a gain in rotor motions at the melting point of the RNAT. The ordinate intercept *y*_0_ of the EEC can then be regarded as an approximation of the average stacking enthalpy (12,24,66). For hsp17, *y*_0_ amounts to (13.0 ± 1.0) kJ/mol and is of similar magnitude as previous estimates of stacking enthalpies (12,24). Better stacking geometries as a result of abolished fraying near the internal loop (see above) may explain the increased *y*_0_ = (18.9 ± 0.8) kJ/mol observed for hsp17^rep^.

It was proposed that nucleobases joined in an EEC form a folding unit (12,24,66) in which the base pairs cooperatively unfold in a sequential zipper-type mechanism beginning from the terminal ends. Figure 7B shows that base pairs in the RBS of hsp17 form a folding unit detached from base pairs in stem I. CD melting curves revealed that the cooperativity of the RBS release is modulated by the internal loop and also influenced through the stability of stem I trapping part of the start codon. The precise temperature regulation of the RBS release is then determined by non-nearest neighbour effects, which are partly transmitted through the hydration shell, and enables the wt hsp17 RNAT to function as a molecular dimmer. Incorporation of the extremely stable C8-G* base pair joins stems I and II and extends the folding unit up to the base pair A5-U37. Non-nearest neighbour effects couple the stability of G* and G28 thereby forming a stable CG clamp that determines the melting behaviour of hsp17^rep^. Since the entropies of both base pairs are very high, the descent in stability is steep and base-pair stabilities are equalized over a narrow temperature range. Both the elongation of the folding unit and the increased entropy difference of the stable CG clamp are responsible for the significantly higher unfolding cooperativity. In other words, replacing the internal bulge by the stable C8-G* base pair turns the molecular dimmer into a binary switch and hence explains the complete loss of activity observed in biological assays with the hsp17^rep^ mutant (16).

## CONCLUSIONS

In this and previous works from us (12,16,67), we investigated the thermodynamics underlying the melting behaviour of two representatives of short hairpin RNATs: the cyanobacterial hsp17 and the *Salmonella agsA* fourU RNAT. In hsp17, an unstructured 10 nt loop separates the RBS and the anti-RBS sequence. Two cytosine residues in the anti-RBS motif stabilize the helix sequestering the RBS by forming stable Watson–Crick base pairs with the guanines in the RBS. In the *agsA* RNAT, the two cytosines are replaced by uridine residues in the anti-RBS motif of the fourU motif. The intrinsic stability loss of the UA and UG pairs is here compensated by a stable GC base pair adjacent to the RBS and a stable tetraloop.

A single mutation was sufficient to shut down both RNATs highlighting the importance of destabilizing mismatches and internal bulges as key elements of the RNAT design. But how can an RNA thermometer benefit from a destabilizing element? One answer is that such an element lowers the melting point of the RNAT to physiological temperatures. Yet, this could also be accomplished by reducing the number of base pairs or altering the GC content as a recent example of a minimal fourU *shuA* RNAT in pathogenic *Shigella dysenteriae* shows (14). While the *shuA* thermometer and other virulence-related thermosensors need to respond to a defined temperature of 37°C, heat shock thermometers such as the ones upstream of the *hsp17* or *agsA* genes need to modulate expression in response to wide range of ambient temperatures in order to titrate the cellular amount of small heat shock proteins according to the cellular demand. We found for both RNATs that base pairs within the RBS are coupled via EEC. The cooperativity of the resulting folding unit determines the number of accessible ribosome binding sites at a given temperature. The melting point of the hsp17 RNAT exceeds the physiological temperature range of *Synechocystis*. This guarantees that increasing heat can always be met with a nonlinear (exponential) switching response yielding sufficient expression of the Hsp17 chaperone. The same holds also true for the *agsA* RNAT (12). On the contrary, a switch like fourU RNAT recently found in pathogenic *Yersinia* (2,4) has a melting point (*T*_m_ = 33°C, unpublished data) that is in the middle of the physiological temperature range. As confirmed by temperature-dependent expression studies (2,4), the melting behaviour of the RNAT avoids expression of a critical virulence factor at temperatures encountered by *Yersinia* under free-living conditions (68), while expression of the virulence factor is nearly maximal at human body temperature. Our results consistently show that the internal loop and the mismatch lower the unfolding cooperativity of the RBS release and equip the hsp17 and the *agsA* RNATs to function as a molecular dimmer. These results may further explain the frequent appearance of destabilizing elements in naturally occurring RNATs that control the expression of heat shock proteins.

Synthetic biology approaches developed empirical guidelines based on naturally occurring RNAT motifs to create a variety of functional RNATs with specific regulatory properties (69,70). Yet, the unexpected behaviour of many *in silico* designed sequences necessitates experimental screening and additional directed evolution approaches (69,71). The poor predictive success may in part be explained by our findings: non-nearest neighbour effects led to an unexpected remote stabilization and extension of the cooperative folding unit within hsp17^rep^ resulting in a strikingly altered response of the RNAT. These effects presumably originate from a thermodynamic coupling of the RNA and the hydration shell. Most of the non-nearest neighbour effects are not accounted for by *in silico* predictions. In fact, the precise interplay of interactions among the nucleobases and the hydration shell is poorly understood and even the most advanced QM/MD simulations cannot reliably predict thermodynamic parameters of the RNA stability at the molecular level (62,64,72).

Many RNATs are substantially more complex than expected from comparison with synthetic RNATs (71) raising the question of whether the model of temperature-controlled RBS release is too simple. For the 127 nt long *prfA* thermometer (3) a second regulatory mechanism was identified: a truncated SAM riboswitch binds to the *prfA* RNAT and inhibits expression of the virulence regulator PrfA (73) showing that at least RNATs are prone to *trans* regulation. Recently, Reining *et al*. showed (74) that the ligand binding affinity of an *addA* riboswitch is maintained over a wide range of temperatures by coupling the riboswitch function with a temperature-sensing helix located within the same RNA. In light of these findings, the here observed remote stabilization may have interesting implications: is it possible that RNATs can be modulated by yet undetected switchable remote effects? *Trans*-acting molecules, such as metabolites or even small ncRNA, could influence the stability at the binding site and remotely (de)stabilize the RBS in the trapped conformation or lead to slippage-like reordering of helices.

## SUPPLEMENTARY DATA

Supplementary Data are available at NAR Online.

SUPPLEMENTARY DATA
