# Supplementary material for: Mechanistic insights into temperature-dependent regulation of the simple cyanobacterial hsp17 RNA thermometer at base-pair resolution
Source: Nucleic Acids Res. 2015 May 4;43(11):5572–85. doi: 10.1093/nar/gkv414 (PMC4477652; doi:10.1093/nar/gkv414)
Supplement: SUPPLEMENTARY DATA [file supp_43_11_5572__index.html]

Mechanistic insights into temperature-dependent regulation of the simple cyanobacterial hsp17 RNA thermometer at base-pair resolution — Mechanistic insights into temperature-dependent regulation of the simple cyanobacterial hsp17 RNA thermometer at base-pair resolution — SUPPLEMENTARY DATA 

# Mechanistic insights into temperature-dependent regulation of the simple cyanobacterial hsp17 RNA thermometer at base-pair resolution

## SUPPLEMENTARY DATA

**Files in this Data Supplement:**

- SUPPLEMENTARY DATA
