# Supplementary material for: Mechanistic insights into temperature-dependent regulation of the simple cyanobacterial hsp17 RNA thermometer at base-pair resolution
Source: Nucleic Acids Res. 2015 May 4;43(11):5572–85. doi: 10.1093/nar/gkv414 (PMC4477652; doi:10.1093/nar/gkv414)
Supplement: SUPPLEMENTARY DATA [file supp_gkv414_nar-00020-r-2015-File009.pdf]

Supplementary information

Mechanistic insights into temperature-dependent regulation of the simple cyanobacterial hsp17 RNA thermometer at base pair-resolution

Dominic Wagner[1], Jörg Rinnenthal[1], Franz Narberhaus[2] and Harald Schwalbe[1]*

[1] Institute for Organic Chemistry and Chemical Biology, Center for Biomolecular Magnetic Resonance, Johann Wolfgang Goethe-University, Max-von-Laue-Strasse 7, D-60438 Frankfurt/Main, Germany.

[2] Microbial Biology, Ruhr University, Universitätsstr. 150, D-44780 Bochum (Germany)

* To whom correspondence should be addressed. Tel: ++49 69 7982 9737;

Email: Schwalbe@nmr.uni-frankfurt.de

Materials and Methods

Table S 1-1. DNA and RNA sequence of the hsp17 RNA thermometer

DNA		RNA	
**5'-**	GAATTCTAATACGACTCACTATA**GGGTAAT**	**5'-**	**ggguaaucaauuccuuccacacaucaggag**
	**CAATTCCTTCCACACATCAGGAGTTAACAT**		**uuaacauuaua**accggaugugcuuuccggu
	**TATA**ACCGGATGTGCTTTCCGGTCTGATGA		cugaugaguccgugaggacgaaauaauguu
	GTCCGTGAGGACGAAATAATGTTAACGGAT		aacggauc **-3'**
	CC **-3'**		

5‘ and 3‘ located endonuclease restriction sites used for subcloning of the gene into the high copy plasmid puc57: *EcoRI*, *BamHI*. Underlined: M9 promoter, bold: hsp17 RNA thermometer, grey and blue: hammerhead ribozyme and hsp17 complementary sequence. The mass of the 41 nt hsp17 RNA construct is 12.9 kDa.

Table S 1-2. DNA and RNA sequence of the hsp17^{rep} RNA thermometer

DNA		RNA	
**5'-**	GAATTCTAATACGACTCACTATA**GGGTAAT**	**5'-**	**ggguaaucaauuccuuccacacaucaggag**
	**CAATTCCTTCCACACATCAGGAGTTGATTA**		**uugauuaua**accggaugugcuuuccggucu
	**TA**ACCGGATGTGCTTTCCGGTCTGATGAGT		gaugaguccgugaggacgaaauaaucaacg
	CCGTGAGGACGAAATAATCAACGGATCC **-3'**		gauc **-3'**

5‘ and 3‘ located endonuclease restriction sites used for subcloning of the gene into the high copy plasmid puc57: *EcoRI*, *BamHI*. Underlined: M9 promoter, bold: hsp17^{rep} RNA thermometer, grey and blue: hammerhead ribozyme and hsp17^{rep} complementary sequence. The mass of the 39 nt hsp17^{rep} RNA construct is 12.3 kDa.

Table S 1-3. DNA and RNA sequence of the hsp17^{stab} RNA thermometer

DNA		RNA	
**5'-**	GAATTCTAATACGACTCACTATA**GGGTAAT**	**5'-**	**ggguaaucaauuccuuccacacaucaggag**
	**CAATTCCTTCCACACATCAGGAGTTAACAT**		**uuaacauuauccc**gggucggcauggcaucuc
	**TATCC**GGGTCGGCATGGCATCTCCACCTCC		caccuccucgcgguccgaccugggcuacuu
	TCGCGGTCCGACCTGGGCTACTTCGGTAGG		cgguaggcuaagggagaaggauc **-3'**
	CTAAGGGAGAAGGATCC **-3'**		

5‘ and 3‘ located endonuclease restriction sites used for subcloning of the gene into the high copy plasmid puc57: *EcoRI*, *BamHI*. Underlined: M9 promoter, bold: hsp17^{stab} RNA thermometer, grey: HDV ribozyme. The mass of the 42 nt hsp17^{stab} RNA construct is 13.5 kDa.

Table S 2. Experimental parameters of the NMR spectroscopic assignment experiments of $hsp17^{rep}$ and $hsp17^{stab}$. F1 and F2 denote indirectly and directly sampled dimension.

	$hsp17^{rep}$				$hsp17^{stab}$			
Experiment	^{1}H-^{1}H NOESY		HNN-COSY		^{1}H-^{1}H NOESY		HNN-COSY	
Aquistion time (F1, F2)	45 ms	70 ms	15.8 ms	70 ms	18 ms	220 ms	35.5 ms	150 ms
Spectral width (F1, F2)	12.4 ppm	20.0 ppm	100 ppm	20.0 ppm	12.0 ppm	19.5 ppm	120 ppm	20 ppm
Offset (F1, F2)	8.5 ppm	4.7 ppm	180 ppm	4.7 ppm	8.5 ppm	4.7 ppm	190 ppm	4.7 ppm
Field strength	21.1 T		18.8 T		22.3 T		14.1 T	
Temperature	273 K		273 K		284 K		284 K	
Sample concentration	600 µM		600 µM		550 µM		400 µM	
Labeling	^{15}N		^{15}N		natural abundance		^{15}N	
Buffer composition	5 mM $K_xH_yPO_4$, pH 6.5, 8 mM KCl, 10 % D_2O		5 mM $K_xH_yPO_4$, pH 6.5, 8 mM KCl, 10 % D_2O		15 mM $K_xH_yPO_4$, pH 6.5, 25 mM KCl, 10 % D_2O		5 mM $K_xH_yPO_4$, pH 6.8, 245 mM KCl, 10 % D_2O	

DSS as internal temperature reference

The chemical shifts of non-labile protons are (to a good approximation) insensitive to temperature. On the contrary, the chemical shifts of water protons/deuterons are strongly affected by temperature. Since the lock system uses the deuterium resonance frequency to keep the magnetic field strength of the NMR spectrometer constant, changes in temperature lead to an apparent change of the chemical shifts of non-labile protons. This fact can be exploited to determine the temperature within the NMR sample from the methyl protons of the internal NMR standard DSS. We determined the internal temperature calibration as follows:

A ^{1}H-NMR spectrum (field strength 600.13 MHz) was recorded of a temperature equilibrated perdeuterated methanol sample. The temperature within the NMR sample at the given airflow heater settings was calculated from the difference of the chemical shifts of the labile proton and the methyl protons as described by Findeisen & Berger. The methanol sample was replaced by the NMR sample of $hsp17^{rep}$, 100 mM $K_xH_yPO_4$, pH 6.8, 120 mM KCl, 10 % D_2O, 100 µM DSS. Meanwhile the airflow heater setting was not altered. After temperature equilibration, the NMR spectrometer was locked on D_2O and the lock parameters were optimized. A ^{1}H-NMR spectrum was recorded and referenced to the methyl protons of DSS. This procedure was repeated for temperatures ranging from 275 K to 325 K. The referencing parameter (SR_{DSS}-value) was fitted to the temperature using a quadratic function (Figure S1).

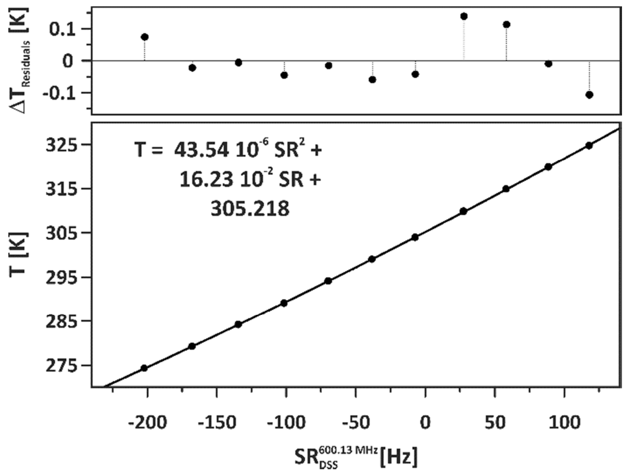

Figure S1. Internal temperature calibration based on the SR_{DSS} value at a field strength of 600.13 MHz.

Findeisen and Berger estimate the error of the temperature obtained from perdeuterated methanol to be 0.2 K. Since the SR value depends on the lock phase, the error of the temperature obtained from the SR_{DSS} value is increased. We found, that small variations of the lock phase (± 15°) result in changes of the SR_{DSS} value of not more than 1.0 Hz. We estimated the uncertainty of the temperature to be 0.3 K by error propagation.

Calculation of the reaction rate $k_{Tr,ext}$

Assuming that the transition state of the externally catalysed proton transfer in the open state is resembled by imino proton exchange of the free nucleotides, the reaction rate $k_{Tr,ext}$ of the external catalysis can be calculated from the exchange rates $k_{ex}^{UTP,GTP}$ of the free nucleotides in the presence of the catalyst HPO_4^{2-}

$$k_{Tr,ext}(T)_{c_{HPO_4^{2-}}} = \frac{1+\sqrt[3]{M_c/M_{RNA}}}{1+\sqrt[3]{M_c/M_{UTP,GTP}}} * k_{ex}^{UTP,GTP}(T)_{c_{HPO_4^{2-}}} \qquad \text{(eq. S1)}$$

Here a correction factor was introduced to account for the differences in diffusion of the mononucleotide and the RNA (1). M_c, $M_{UTP,GTP}$ and M_{RNA} are the molecular masses of the catalyst HPO_4^{2-}, the mononucleotide and the RNA. At 20°C the exchange rates were reported as $k_{ex,c_{HPO_4^{2-}}=1\,mM}^{UTP} = (2032 \pm 69)\ \mathrm{Hz}$ and $k_{ex,c_{HPO_4^{2-}}=1\,mM}^{GTP} = (1563 \pm 36)\ \mathrm{Hz}$ and the temperature

dependence of $k_{ex}^{UTP,GTP}$ was previously determined at a concentration of the catalyst HPO_4^{2-} $c_{HPO_4^{2-}} = 1\ mM$ (2):

$$k_{ex}^{UTP,GTP}(T)_{c_{HPO_4^{2-}}=1\,mM} = \frac{k_B T}{h} * \exp\left(-\frac{\Delta H_{Tr}^{UTP,GTP} - T\,\Delta S_{Tr}^{UTP,GTP}}{R\,T}\right) \quad \text{(eq. S2)}$$

in which k_B, h and R are the Boltzmann, the Planck and the universal gas constant, $\Delta H_{Tr}^{UTP} = (39.65\ \pm 3.75)\ \mathrm{kJ\ mol^{-1}}$, $\Delta S_{Tr}^{UTP} = (46.13\ \pm 13.58)\ \mathrm{J\ mol^{-1} K^{-1}}$ and $\Delta H_{Tr}^{GTP} = (34.64\ \pm 2.59)\ \mathrm{kJ\ mol^{-1}}$, $\Delta S_{Tr}^{GTP} = (-65.42\ \pm 9.37)\ \mathrm{J\ mol^{-1} K^{-1}}$. At a higher concentration of the catalyst HPO_4^{2-} the exchange rate can be extrapolated:

$$k_{ex}^{UTP,GTP}(T)_{c_{HPO_4^{2-}}} = c_{HPO_4^{2-}} * k_{ex}^{UTP,GTP}(T)_{c_{HPO_4^{2-}}=1\,mM} \quad \text{(eq. S3)}$$

Monte Carlo simulation

ΔH_{Diss}, ΔS_{Diss}, ΔG_{Diss} and their standard errors $\Delta\Delta H_{Diss}$, $\Delta\Delta S_{Diss}$, $\Delta\Delta G_{Diss}$ were determined by a Monte Carlo (MC) simulation, in which experimental data were repeatedly Gaussian noised and fitted. The purpose of the MC simulation is to account for error propagation of the input parameters with differing errors. The MC simulation was performed in *Mathematica* (3).

PREPARATION OF NOISED DATASET In general, the parameter `var` with exp. error `deltaVar was` Gaussian noised using the function `RandomVariate[NormalDistribution[var,deltaVar]] in` *Mathematica*. Gaussian distributed noise was added to each data point $(T,\ k_{ex})$ that had been obtained for a given nucleobase at a given catalyst concentration. Each data point was weighted using the weight $1/\ \Delta k_{ex}$. The parameters for the external transition state (eq. S3) were also Gaussian noised: here, a relative error ($\Delta c_{relative}$=2%) was added to the concentration of the catalyst $c_{HPO_4^{2-}}$. The noising of the enthalpy ΔH_{Tr}^{NTP} and entropy ΔS_{Tr}^{NTP} of the external transition state was performed such that the resulting rate of $k_{ex,c_{HPO_4^{2-}}=1\,mM}^{NTP}$ (eq. S2) resembles a normal distribution with standard deviation $\Delta k_{ex,c_{HPO_4^{2-}}=1\,mM}^{NTP}$ (errors for all three parameters are given above).

Fitting of Noised Dataset The noised data points including the noised external transition state parameters were fitted to eq. 8 (main text) against the temperature and catalyst concentration using the *Mathematica* function `NonlinearModelFit[]` to obtain the fit parameters given in table S3.

Table S3. Fit parameters and lower and upper restraints used in the fitting procedure

fit parameter	***lower restraint***	***upper restraint***
ΔH_{diss} [kJ mol^{-1}]	10,000	500,000
ΔS_{diss} [J K^{-1}mol^{-1}]	10	1,200
$\Delta H^{Tr,int}$ [kJ mol^{-1}]	10,000	500,000
$\Delta S^{Tr,int}$ [J K^{-1}mol^{-1}]	-300	1,200
d [Hz]	0	12

Here, we used the numerical minimizer “`NMinimize`” as method applying “`DifferentialEvolution`” as global genetic search algorithm followed by a local analytical `"QuasiNewton"` minimization; the following option was given to the `NonlinearModelFit[]` function:

```
fitMethod={
        NMinimize, MaxIterations->400,
        Method->{
                "DifferentialEvolution","SearchPoints"->60,"ScalingFactor"->0.90,
                "CrossProbability"->0.05,
                "PostProcess"->
                        {FindMinimum,Method->"QuasiNewton"}
}};
```

Additionally we employed ample restraints, which are given in table S3. In total 1000 noised datasets were prepared and fitted for each nucleobase.

Evaluation of Fit Results ΔH_{Diss}, ΔS_{Diss}, ΔG_{Diss} at 20°C and $\Delta H^{Tr,int}$, $\Delta S^{Tr,int}$ were calculated as the average of the individual fit results. The respective errors were calculated as the standard deviation as suggested by Kohler *et. al* (4) and denoted as Monte Carlo error. $k_{Tr,int}$ at 20°C was calculated as the geometric mean µ and the error is given as multiplicative standard deviation s, which reads as µ */ s and describes the interval [µ / s, µ * s] containing the fit results of $k_{Tr,int}$ at the one-σ level (5). We noticed that the noising procedure occasionally produced datasets, which resulted in ill-fit minimizations. These outliers were identified based on the fit statistics and excluded from the averaging (tables S4 and S5).

Results

Imino proton exchange rates at a catalyst concentration of 2 mM HPO_4^{2-}

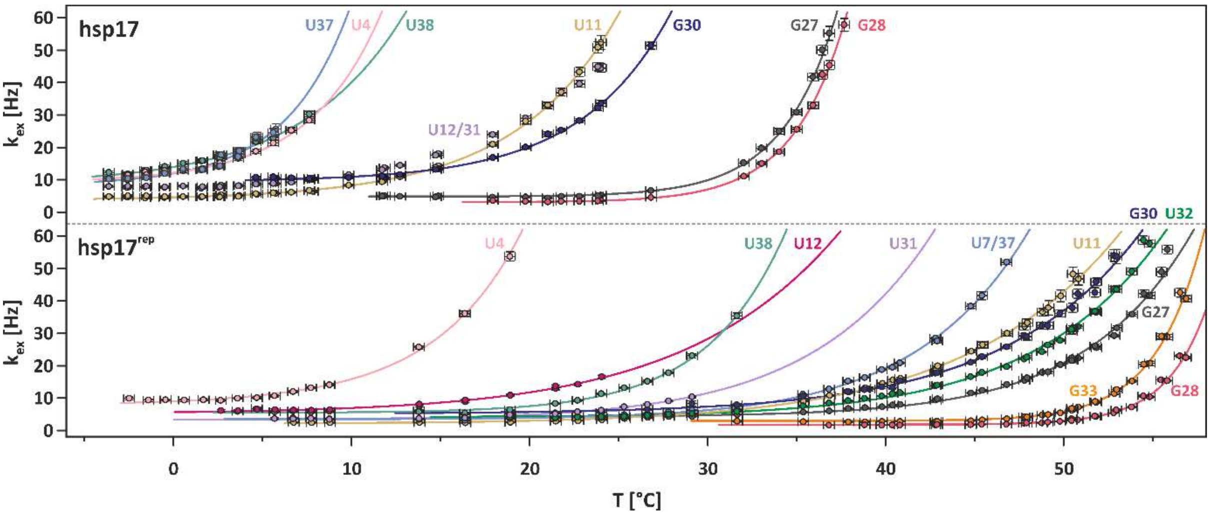

Figure S2. Imino proton exchange rates k_{ex} of indicated nucleobases from hsp17 and hsp17^{rep} at varying temperatures and a catalyst concentration of 2 mM HPO_4^{2-} and interpolations obtained from the Monte Carlo simulation. Error bars represent standard errors.

Fit results of the MC-simulation: internal transition state

Table S4. Fit results of the internal transition state of the shown base pairs in hsp17. $\Delta\Delta H^{Tr,int}$, $\Delta\Delta S^{Tr,int}$ represent the Monte Carlo error and $\Delta k_{Tr,int}$ (T=20°C) represents the multiplicative (dimensionless) error. *No. of acc. fits* denotes the number of accepted fits in the MC-simulation (1000 runs performed).

hsp17	*No. of acc. fits*	$\Delta H^{Tr,int}$ [kJ mol^{-1}]	$\Delta\Delta H^{Tr,int}$ [kJ mol^{-1}]	$\Delta S^{Tr,int}$ [J mol^{-1}K^{-1}]	$\Delta\Delta S^{Tr,int}$ [J mol^{-1}K^{-1}]	$k_{Tr,int}$ (T=20°C) [10^3 Hz]	$\Delta k_{Tr,int}$ (T=20°C)
G3/U40	*n.d.*	*n.d.*	*n.d.*	*n.d.*	*n.d.*	*n.d.*	*n.d.*
U4	1000	114.5	34.2	231.9	122.0	31.2	1.9
U38	964	43.7	16.2	-12.2	57.6	23.5	1.4
U37	993	131.9	43.9	295.8	157.4	53.9	1.0
U7	*n.d.*	*n.d.*	*n.d.*	*n.d.*	*n.d.*	*n.d.*	*n.d.*
U32	*n.d.*	*n.d.*	*n.d.*	*n.d.*	*n.d.*	*n.d.*	*n.d.*
U31	/	/	/	/	/	/	/
U11	1000	*99.7*	7.1	168.4	24.1	6.5	1.1
G30	1000	109.9	10.8	204.1	36.3	7.3	1.1
U12	/	/	/	/	/	/	/
G28	930	77.1	32.8	96.3	106.8	11.9	1.9
G27	986	117.1	30.9	226.3	100.8	5.4	1.8
U15	*n.d.*	*n.d.*	*n.d.*	*n.d.*	*n.d.*	*n.d.*	*n.d.*

n.d. = not determinable due to low base pair stability and resultant exchange broadening of the imino resonance. U31 and U12 could not be analysed due to spectral overlap.

Table S5. Fit results of the internal transition state of the shown base pairs in hsp17^{rep}. $\Delta\Delta H^{Tr,int}$, $\Delta\Delta S^{Tr,int}$ represent the Monte Carlo error and $\Delta k_{Tr,int}$ (T=20°C) represents the multiplicative (dimensionless) error. *No. of acc. fits* denotes the number of accepted fits in the MC-simulation (1000 runs performed). Nucleobase numbering of hsp17^{rep} is based on hsp17, G* denotes the mutation AAC(33-35)G.

**hsp17^{rep}**	***No. of acc. fits***	**$\Delta H^{Tr,int}$** [kJ mol^{-1}]	**$\Delta\Delta H^{Tr,int}$** [kJ mol^{-1}]	**$\Delta S^{Tr,int}$** [J mol^{-1}K^{-1}]	**$\Delta\Delta S^{Tr,int}$** [J mol^{-1}K^{-1}]	**$k_{Tr,int}$ (T=20°C)** [10^{3}Hz]	**$\Delta k_{Tr,int}$ (T=20°C)**
G3/U40	*n.d.*	*n.d.*	*n.d.*	*n.d.*	*n.d.*	*n.d.*	*n.d.*
U4	999	112.1	18.8	211.3	64.6	7.1	1.3
U38	999	89.3	18.7	124.3	62.4	2.3	1.3
U7/U37	997	46.7	10.7	-3.1	34.5	20.1	1.3
G*	827	65.6	31.4	41.9	96.3	2.0	3.4
U32	1000	52.2	5.4	9.8	17.5	9.9	1.2
U31	886	40.5	14.9	-17.4	50.1	46.1	1.2
U11	1000	63.2	4.9	36.8	16.0	2.8	1.2
G30	998	76.2	5.7	75.1	18.4	1.4	1.2
U12	924	52.7	10.9	26.3	37.4	59.4	1.1
G28	1000	187.5	40.4	413.3	123.3	0.0092	5.8
G27	1000	59.0	7.2	22.1	22.6	2.7	1.3
U15	*n.d.*	*n.d.*	*n.d.*	*n.d.*	*n.d.*	*n.d.*	*n.d.*

n.d. = not determinable due to low base pair stability and resultant exchange broadening of the imino resonance.

Enthalpy-entropy compensation (EEC)

STATISTICAL COMPENSATION EFFECT The statistical compensation effect is a correlation of indirectly measured parameters (e.g. ΔH_{diss}, ΔS_{diss} obtained from van't Hoff analysis) that arises as a result of experimental uncertainties and error compensation of additive parameters with opposing sign (6, 7). A *phantom* EEC is likely to be observed for interactions with similar Gibbs energies and if ΔH_{diss}, ΔS_{diss} values were obtained over a comparable temperature range and without independent measurements of the correlated parameters (8). A characteristic of the statistical compensation effect is that the slope of the correlation approximately equals the harmonic mean of the temperatures T_{hm} used to derive the correlation (6, 9).

STATISTICAL TEST The statistical significance of the observed EEC (figure 5, main article) can be probed by the following relation (9, 10):

$$T_{hm} - 2\sigma_m < m_{EEC} < T_{hm} + 2\sigma_m \qquad \text{(eq. S4)}$$

Here, the m_{EEC} and σ_m denote the slope and its standard deviation of the observed EEC. T_{hm} is the weighted harmonic mean of the experimental temperatures T_i, which were included in the determination of all ΔH_{diss}- and ΔS_{diss}-values defining the EEC:

$$T_{hm} = \frac{\sum w_i}{\sum {w_i}/{T_i}} \qquad \text{(eq. S5)}$$

In eq. S5, the same weights w_i were used as in the MC simulation. If the slope of the EEC fulfils inequality eq. S4, the EEC is likely to arise from the statistical compensation effect (6) and the observed correlation should be interpreted as insignificant, otherwise the correlation is considered to be significant.

For hsp17 and hsp17^{rep}, the weighted harmonic means were calculated as T_{hm} = 288.9 K and T_{hm} = 305.1 K, respectively. The fit results of the EEC were m_{EEC} = (322.5 ± 2.7) K for hsp17 and m_{EEC} = (334.7 ± 2.3) K for hsp17^{rep}. Clearly, the slopes m_{EEC} lie with deviations of 12.4 σ_m and 12.9 σ_m well outside the interval $[T_{hm} - 2\sigma_m, T_{hm} + 2\sigma_m]$ for both RNATs and cannot fulfil eq. S4. Therefore the EEC shown in figure 5 must be considered significant.

Global unfolding of hsp17^{stab}

A

$$a(T) = \frac{1 + \tanh\left(\frac{\Delta H_{unf}}{2R}\left(T_m^{-1} - T^{-1}\right)\right)}{2}$$

B

$$CD(T) = (1 - a(T)) * Cd_{folded}(T) + a(T) * Cd_{unfolded}(T)$$

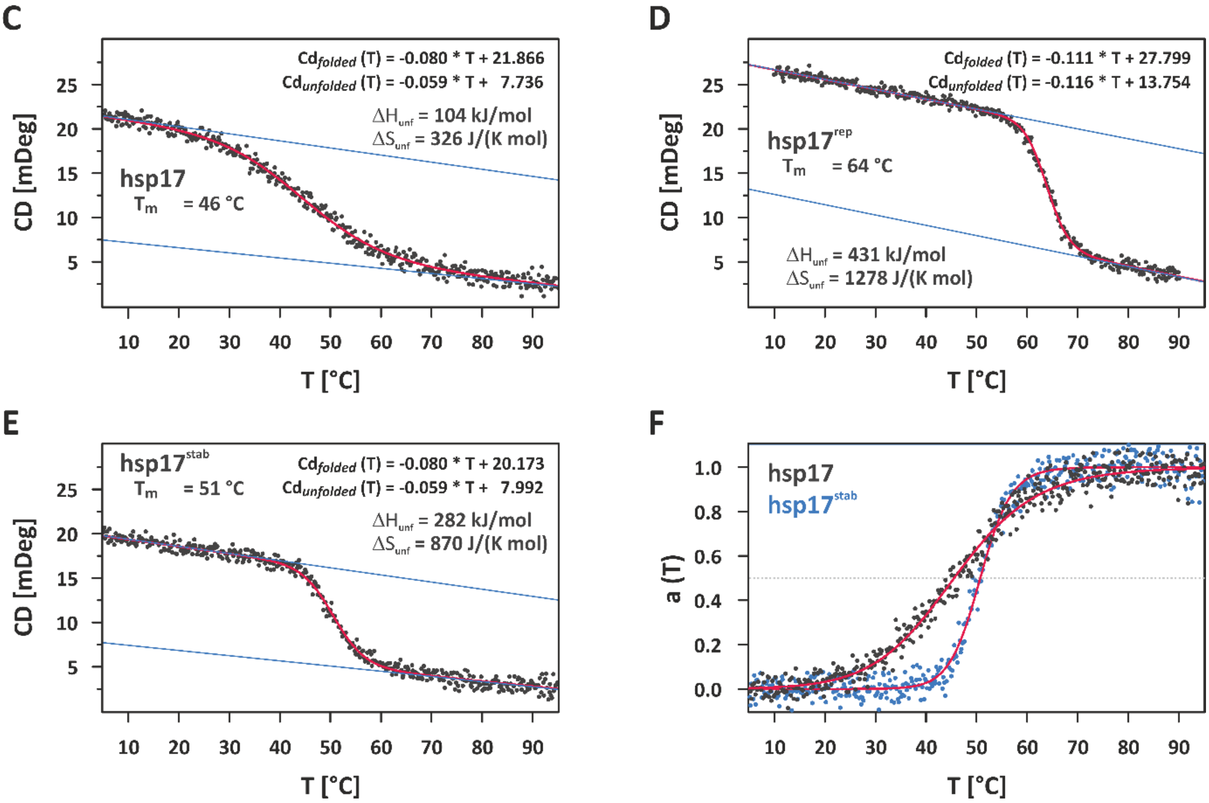

Figure S3. (A) The fraction unfolded $a(T)=n_{open}/(n_{open}+n_{closed})$ as a function of the temperature T can be expressed in terms of the unfolding enthalpy ΔH_{unf} and the melting point T_m of the RNA hairpin by combining the van't Hoff- and the Gibbs-Helmholtz equations (R denotes the universal gas constant). (B) Equation used to fit the CD melting curve of a sigmoidal 2-state unfolding transition: $Cd_{folded}(T)$ and $Cd_{unfolded}(T)$ describe the linear temperature dependence of the CD effect in the folded/unfolded state. (C), (D) and (E) show the analysis of the CD melting curves of hsp17, hsp17^{rep} and hsp17^{stab} assuming a 2-state unfolding transition. The CD-melting curve of hsp17^{stab} was recorded at a sample concentration of c = 10 µM. Data were fitted against equation shown in B): parameters of the CD-baselines were allowed to adjust freely and soft restraints were employed on 32°C<T_m<80°C. Due to its broad temperature response, the baselines $Cd_{folded}(T)$, $Cd_{unfolded}(T)$ of hsp17 could not be defined unambiguously. We therefore fitted the CD melting curves of hsp17 and hsp17^{stab} in a global fit under the assumption, that the baselines of the open and closed state of both RNA hairpins exhibit the same temperature dependence. (F) Fraction unfolded of hsp17 (red) and hsp17^{stab} (blue).

RNA preparation

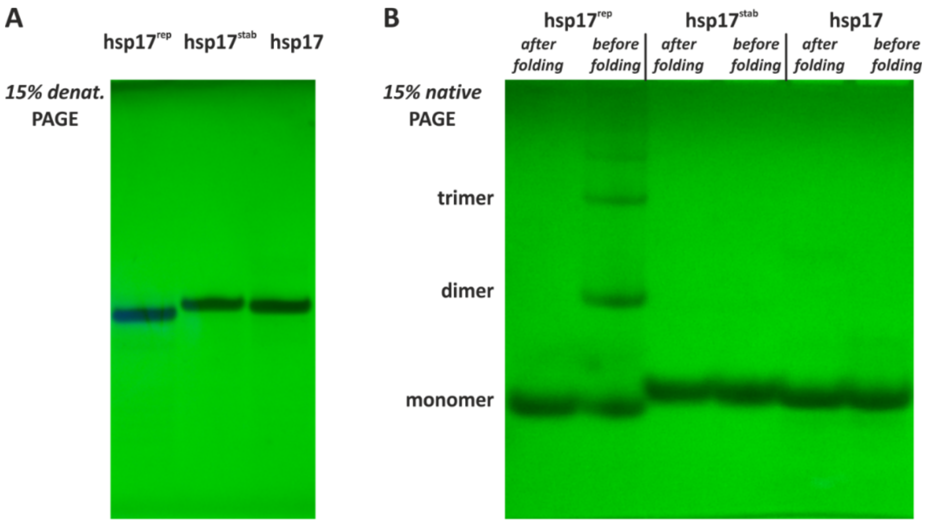

Figure S4. *Polyacrylamide gel electrophoresis* (PAGE) of purified NMR samples under (A) denaturing (7 M Urea) and (B) native conditions visualized by UV-shadowing.

NMR assignment

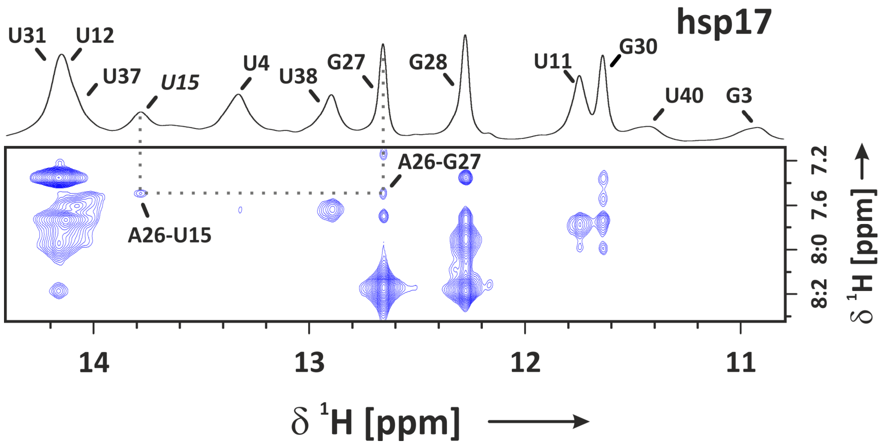

Figure S5. Imino/aromatic region in the ^{1}H-^{1}H-NOESY (bottom) and ^{1}H-NMR (top) spectrum of hsp17. Cross peaks between H2 of A26 and the imino protons of U15 and G27 confirm the imino assignment of U15. The experiment was recorded at a sample concentration of 600 µM and in 5 mM K_xH_yPO4, pH 6.5, 8 mM KCl, 10 % D_2O at T = 275 K, B_0 = 18.1 T and a NOESY mixing time of 50 ms.

Discussion

Individual base pair stability and global unfolding stability

Here, we want to present a brief recapitulation of the thermodynamics that can be probed by CD melting curve analysis and imino exchange spectroscopy of RNA hairpins. We discuss how the results obtained from the different experimental approaches are related to each other.

CD melting curve analysis determines the energy difference of the folded and the unfolded state of an RNA hairpin. The folded state can be considered as an ensemble comprised of RNA molecules residing in a helical conformation (figure S6, left side). Occasionally individual base pairs undergo an opening reaction without perturbing the backbone conformation of the helix.

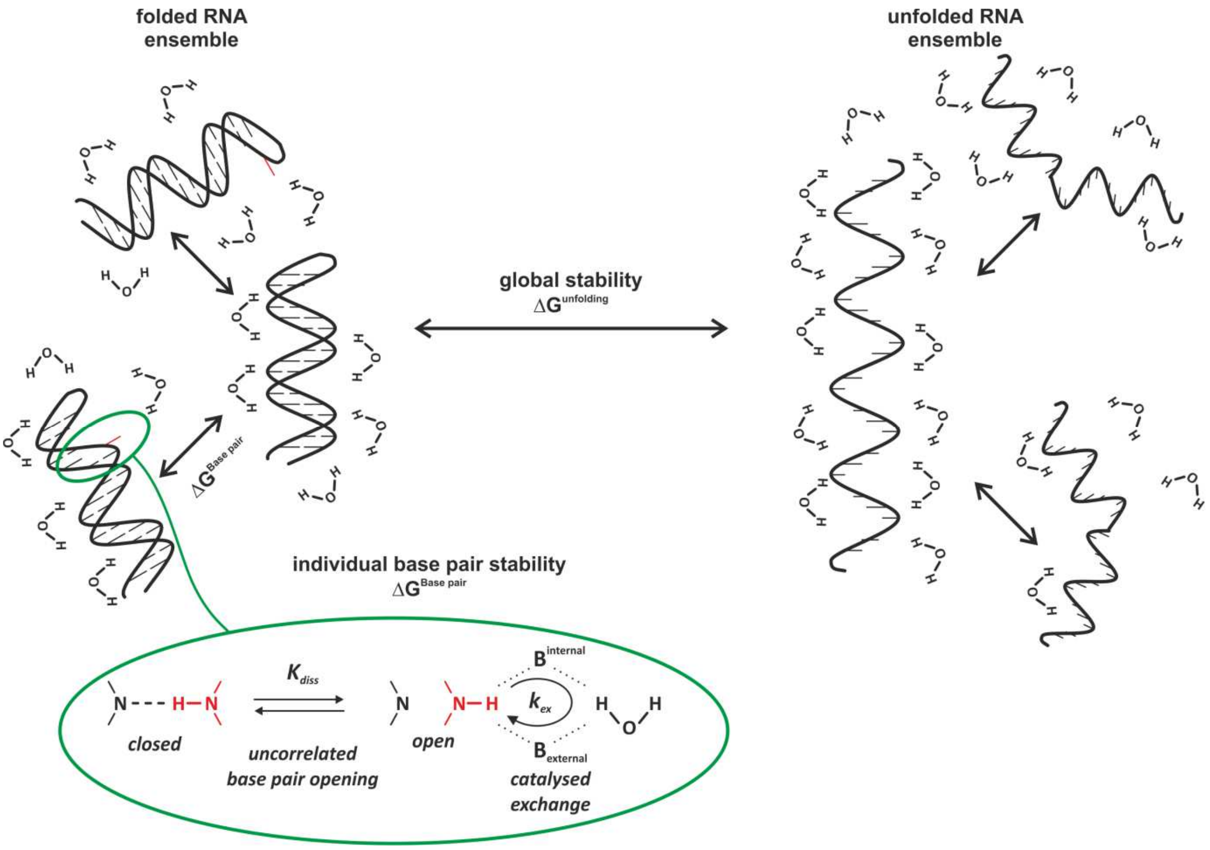

Figure S6. Schematic representation of the dissociation/unfolding reactions investigated in this study. $\Delta G^{Base\ pair}$ can be obtained from imino exchange spectroscopy and represents the free energy of the individual base pair opening events in the the EX2 condition (in the main text denoted as ΔG_{diss}). The stability of the global unfolding can be determined from CD metling curve analysis.

Each ensemble member is solvated by its respective hydration shell. In the unfolded state (figure S6, right side), the ensemble of RNA hairpins exists in a linear conformation with residual stacking interactions among the nucleobases. In this conformation most of the intramolecular hydrogen bonds and stacking interactions are lost. The charge repulsion between phosphate anions in the backbone

of the neighboring strands is relieved compared to the double stranded conformation. Depending on the degree of residual structure, the rotational entropy within the molecule is increased compared to the folded state. As discussed in the manuscript, there is increasing evidence to support our view, that the structure and dynamics of the hydration sphere is very dependent on the RNA conformation. Therefore it is only reasonable to conclude that the interaction between the RNA and the hydration shell is also different in folded and unfolded RNAs.

Imino NMR spectroscopy exploits the fact that hydrogen bonded nucleobases open and close repeatedly reaching an equilibrium which can be probed by imino proton water exchange NMR methodology (figure S6, green inlet). The lifetime of the open state is in the order of ~1 ns (1, 11) and the opening reaction is uncorrelated to other base pair opening events (11, 12). Imino exchange spectroscopy therefore samples energetic contributions from stacking and hydrogen bonds within the base pair and with adjacent water molecules. Additionally entropic contributions from conformational changes of the nucleobases and the hydration sphere influence the stability. Note that, interactions within the charged backbone are not sampled by imino exchange spectroscopy, as the conformation of the backbone is believed to remain fixed during the base pair opening and closing event (13).

Individual base pair stabilities contribute significantly to the global stability of the RNA hairpin. By inspection of tables 1 and 2 it becomes apparent that the sum of Individual base pair stabilities significantly exceeds the global stability of the entire hairpin (figure S3). For example, the free energy of $hsp17^{rep}$ at 20°C amounts to 78 kJ/mol, while the sum of determined base pair stabilities amounts to 297 kJ/mol (note, that 297 kJ/mol is likely to underestimate the overall base pair stabilities by a factor of ~2, as we were only able to determine the stabilities of half of all base pair opening reactions in the $hsp17^{rep}$ RNAT).

How can the apparent overestimation of the global stability by individual base pair stabilities be explained? As described above, imino exchange spectroscopy and CD melting curve analysis both sample the loss of the hydrogen bonds and the ordered stacking interaction of the base pairs. However, the melting of the RNA hairpin is accompanied by structural and dynamical changes such as an altered hydration sphere, reduced charge interactions and increased entropy within the backbone of the unfolded RNA. These effects are sampled by CD spectroscopy but not by imino exchange spectroscopy. Since the thermodynamics of the unfolded state cannot be obtained with current experimental methodology, it is not possible to give a more quantitative comparison of the global stability and the individual base pair stability.

REFERENCES

1. Snoussi, K. and Leroy, J.-L. (2001) Imino Proton Exchange and Base-Pair Kinetics in RNA Duplexes. *Biochemistry*, **40**, 8898–8904.

2. Steinert, H.S., Rinnenthal, J. and Schwalbe, H. (2012) Individual basepair stability of DNA and RNA studied by NMR-detected solvent exchange. *Biophys. J.*, **102**, 2564–74.

3. Wolfram Research, Inc., Champaign, I. (2014). Mathematica, Version 10.0.

4. Koehler, E., Brown, E. and Haneuse, S.J.-P.A. (2009) On the Assessment of Monte Carlo Error in Simulation-Based Statistical Analyses. *Am. Stat.*, **63**, 155–162.

5. Limpert, E., Stahel, W.A. and Abbt, M. (2001) Log-normal Distributions across the Sciences: Keys and Clues. *Bioscience*, **51**, 341.

6. Barrie, P.J. (2012) The mathematical origins of the kinetic compensation effect: 1. The effect of random experimental errors. *Phys. Chem. Chem. Phys.*, **14**, 318–26.

7. Barrie, P.J. (2012) The mathematical origins of the kinetic compensation effect: 2. The effect of systematic errors. *Phys. Chem. Chem. Phys.*, **14**, 327–36.

8. Starikov, E.B. and Nordén, B. (2012) Entropy-enthalpy compensation may be a useful interpretation tool for complex systems like protein-DNA complexes: An appeal to experimentalists. *Appl. Phys. Lett.*, **100**, 193701.

9. Sharp, K. (2001) Entropy-enthalpy compensation: fact or artifact? *Protein Sci.*, **10**, 661–7.

10. Krug, R.R., Hunter, W.G. and Grieger, R.A. (1976) Statistical interpretation of enthalpy–entropy compensation. *Nature*, **261**, 566–567.

11. Guéron, M. and Leroy, J.L. (1995) Studies of base pair kinetics by NMR measurement of proton exchange. *Methods Enzymol.*, **261**, 383–413.

12. Russu, I.M. (2004) Probing site-specific energetics in proteins and nucleic acids by hydrogen exchange and nuclear magnetic resonance spectroscopy. *Methods Enzymol.*, **379**, 152–75.

13. Rinnenthal, J., Klinkert, B., Narberhaus, F. and Schwalbe, H. (2010) Direct observation of the temperature-induced melting process of the Salmonella fourU RNA thermometer at base-pair resolution. *Nucleic Acids Res.*, **38**, 3834–47.
